# Ammonia-Assisted Chemical Vapor Deposition Growth of Two-Dimensional Conjugated Coordination Polymer Thin Films

**DOI:** 10.1021/jacs.5c04515

**Published:** 2025-05-15

**Authors:** Jinxin Liu, Shuai Fu, Yubin Fu, Yunxu Chen, Kian Tadayon, Mike Hambsch, Darius Pohl, Ye Yang, Alina Müller, Fengxiang Zhao, Stefan C. B. Mannsfeld, Lei Gao, Mischa Bonn, Xinliang Feng, Renhao Dong

**Affiliations:** a 28286 Max Planck Institute for Microstructure Physics, Halle (Saale) 06120, Germany; b Center for Advancing Electronics Dresden and Faculty of Chemistry and Food Chemistry, TUD Dresden University of Technology, Dresden 01067, Germany; c 28308 Max Planck Institute for Polymer Research, Mainz 55128, Germany; d Fraunhofer Institute for Ceramic Technologies and Systems (IKTS), Dresden 01109, Germany; e Center for Advancing Electronics Dresden (CFAED) and Faculty of Electrical and Computer Engineering, TUD Dresden University of Technology, Dresden 01062, Germany; f Dresden Center for Nanoanalysis (DCN), Center for Advancing Electronics Dresden (CFAED), TUD Dresden University of Technology, Dresden 01069, Germany; g Department of Chemistry, 25809 The University of Hong Kong, Hong Kong 999077, China; h Materials Innovation Institute for Life Sciences and Energy (MILES), HKU-SIRI, Shenzhen 518048, China

## Abstract

As emerging electroactive materials, the controlled synthesis of highly ordered two-dimensional (2D) conjugated coordination polymer (c-CP) films ensuring the long-range π-electron delocalization is essential for advancing high-performance (opto-)­electronics. Here, we demonstrate the growth of highly crystalline 2D c-CP thin films on inert substrates by chemical vapor deposition with the assistance of ammonia (NH_3_) for the first time, leveraging its deprotonation effect on ligands and competing effect as additional coordinating species. The resulting Fe-HHB (HHB = hexahydroxybenzene) films exhibit large-area uniformity and a 2 order-of-magnitude increase in crystal grain size, which translates into significant improvements in electrical conductivity (from 0.002 to 3 S/cm), charge mobility, elastic modulus, and hardness. To verify the generality of this NH_3_-assisted synthesis, the contrast Cu-HHB and Cu-BHT (BHT = hexathiolbenzene) 2D c-CP thin films are also prepared and deliver significantly improved electrical conductivities from 51 to 113 and from 595 to 905 S/cm, respectively. The greatly improved crystallinity, combined with the high compatibility of the developed synthetic strategy with current device integration technologies, paves the way for developing c-CP-based electronics.

## Graphical abstract

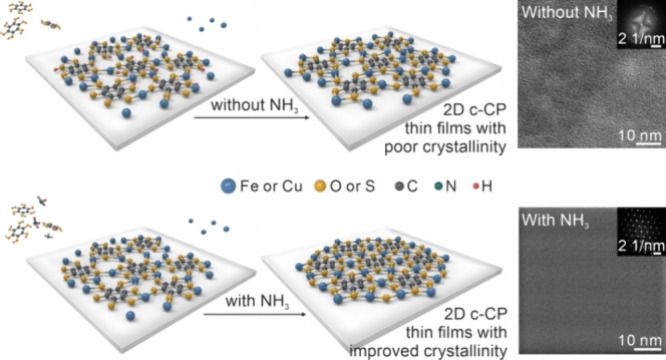

## Introduction

Electrically conductive coordination polymers (ECCPs) and metal–organic frameworks (MOFs) are emerging electroactive materials that have garnered increasing attention in (opto-)­electronics and spintronics.^1–3^ Benefiting from the in-plane π-extended conjugation and out-of-plane π–π interactions, two-dimensional (2D) conjugated CPs (c-CPs) exhibit many intriguing properties,^4,5^ including excellent electrical conductivities (up to 10^3^ S cm^–1^),^6^ remarkable charge mobility (up to 10^2^ cm^2^ V^–1^ s^–1^),^7^ superconductivity,^8^ high photosensitivity,^9^ and intrinsic ferromagnetic ordering.^10^ These characteristics make them potential active layers for a wide range of electronic applications. As a prerequisite for their practical applications, achieving the rational synthesis of high-crystallinity 2D c-CP thin films that allow for the long-range π-electron delocalization and scalable device integration is crucial but remains a major challenge for conventional solution-based approaches.^11^

Reassembly of delaminated 2D c-CP nanosheets enabled straightforward mass production of their thin films but suffered from poorly controlled crystallinity and thicknesses.^11^ To date, most 2D c-CP thin films utilized in fundamental studies and electronic applications have been synthesized at the liquid–liquid or gas–liquid interfaces, which enabled the large-scale production of film samples with variable thicknesses.^5,12^ However, homogeneous nucleation of crystals in solutions generally led to particle contamination,^13,14^ which could compromise the charge transport properties due to the extrinsic charge scattering, thereby restricting device performance. In addition, solution-induced corrosion and surface-tension effects further hindered the integration of the synthetic materials into nanodevices.^15^ Chemical vapor deposition (CVD) could offer a solvent-free approach to ensure the formation of uniform thin films with a residual-free surface and atomic-level conformality.^16^ Featured by large-area deposition and high controllability of crystal growth,^17,18^ the incorporation of CVD and 2D c-CP thin films facilitates seamless compatibility with existing microfabrication technologies,^19,20^ positioning it as an ideal methodology to advance the transition of 2D c-CPs into industrial electronics. Pioneering success was achieved in the growth of crystallized Cu-BHT (BHT = hexathiolbenzene) thin films with a defect-engineered semiconductor–conductor transition^21^ and crystallized Cu-THQ (THQ = tetrahydroxy-1,4-benzoquinone) thin films with a unique edge-on orientation.^22,23^ Thereafter, the growth of patterned Cu-BHT films^19^ and 2D c-CP thin films with ultrasmooth surfaces^20^ further enhanced the compatibility with device integration. Despite the significant advances in the CVD synthesis of c-CP films, the realization of high crystallinity with large-sized crystalline domains has remained a great challenge, which also exerts a substantial impact on device performance.

Herein, we report the controlled CVD growth of high-quality 2D c-CP films on inert substrates using ammonia (NH_3_) gas to aid in the deprotonation of ligands. The residual protons would act as electrostatic hindrances and obstruct the assembly of organized coordination networks,^24^ ultimately resulting in low-crystallinity 2D c-CP films (Figure 1a). Conversely, deprotonation facilitated by the presence of NH_3_ would favor the orderly assembly of precursors, leading to thin films with significantly improved crystallinity (Figure 1b). In addition, NH_3_ can also act as an additional coordinating species to compete with metal–ligand interactions, promoting the reversible formation and breakage of the coordination bonds, thereby leading to high-quality 2D c-CPs. Considering the high dissociation energy of the Fe–O bond (Table S1), Fe-HHB (HHB = hexahydroxybenzene) is selected as the most ideal prototype material to maximize the competing effect of NH_3_. Compared to the one synthesized in the absence of NH_3_ (named **Fe-HHB-o**), the resulting thin films grown in the presence of NH_3_ (named as **Fe-HHB-w**) exhibit an ∼2 order-of-magnitude increase in the grain area (increased from ∼10^2^ to ∼10^4^ nm^2^), leading to substantial improvements in conductivity (from 0.002 to 3 S/cm), mobility (from ∼12 to ∼31 cm^2^/(V·s)), elastic modulus (from ∼25.7 to ∼43.8 GPa), and hardness (from ∼0.9 to ∼2.0 GPa). Moreover, the electrical conductivities of the CVD Cu-HHB and Cu-BHT thin films were increased from ∼51 and ∼595 to ∼113 and ∼905 S/cm, respectively, after adopting the NH_3_-assisted strategy. The contrast results confirm that the NH_3_-assisted synthesis can be applicable to the preparation of various 2D c-CP thin films with improved quality, validating the universality of the proposed strategy.

**Figure 1:**
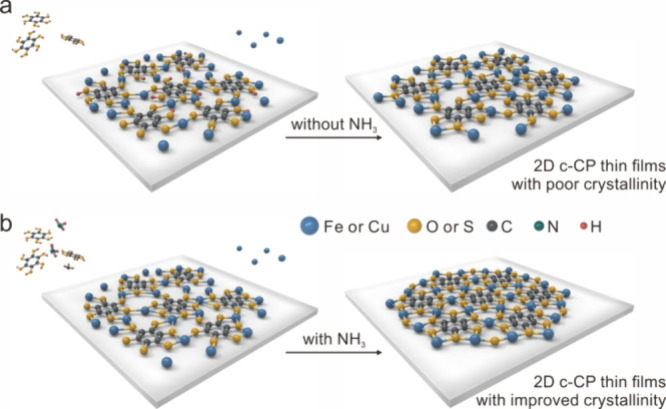
Schematic illustration of (a) the CVD growth process on an inert substrate without NH_3_, resulting in 2D c-CP thin films with poor crystallinity and (b) the NH_3_-assisted growth process, resulting in thin films with highly improved crystallinity. Here, the structure of Fe-HHB is used as the prototype to illustrate the growth strategy.

## Results and Discussion

The CVD growth of **Fe-HHB-w** was performed on SiO_2_/Si or quartz substrates based on an optimal face-to-face inner tube system^21,22^ (Figure 2a and Figure S1), while the sample synthesized without this face-to-face system shows inferior quality (Figure S2). Tris­(acetylacetonato) iron­(III) (Fe­(acac)_3_) and HHB powders were employed as precursors (Figure 2b), and a container of 0.14 mol/L NH_3_·H_2_O was placed upstream, with a small opening to allow the steady release of NH_3_ gas into the chamber under low pressure (∼0.5 mbar). The as-grown **Fe-HHB-w** thin films exhibit visually uniform reflectivity across the ∼1 cm^2^ substrate areas, as evidenced by the macroscopic images (Figure 2c). The optical microscopy (OM) and scanning electron microscopy (SEM) images (Figure 2d and Figure S3) at low magnification indicate uniform contrast across the entire covered region, further affirming the uniform feature of the films. The uniform elemental distribution was also demonstrated by SEM-based energy-dispersive X-ray spectroscopy (EDX) elemental mapping (Figure 2e). In addition, no obvious signal assigned to the N element was detected in the EDX spectra (Figure S4), suggesting that no unintended doping occurred from NH_3_. As revealed by atomic force microscopy (AFM), a continuous uniform Fe-HHB thin film with a thickness of ∼11.5 nm was formed after a reaction time of 10 min (Figure 2f), which further increased to ∼633.2 nm after a 6 h growth with the surface roughness *R*_q_ value reaching ∼8 nm (Figures S5 and S6). Raman spectroscopy was utilized to characterize the coordination information on the synthesized **Fe-HHB-w** film. The disappearance of the signal assigned to the O–H bond at ∼3300 cm^–1^ suggests the efficient coordination between Fe and substitution groups in the presence of NH_3_ (Figure S7).^25^ The homogeneous Raman intensity mapping also verifies the structural uniformity of the film.

**Figure 2:**
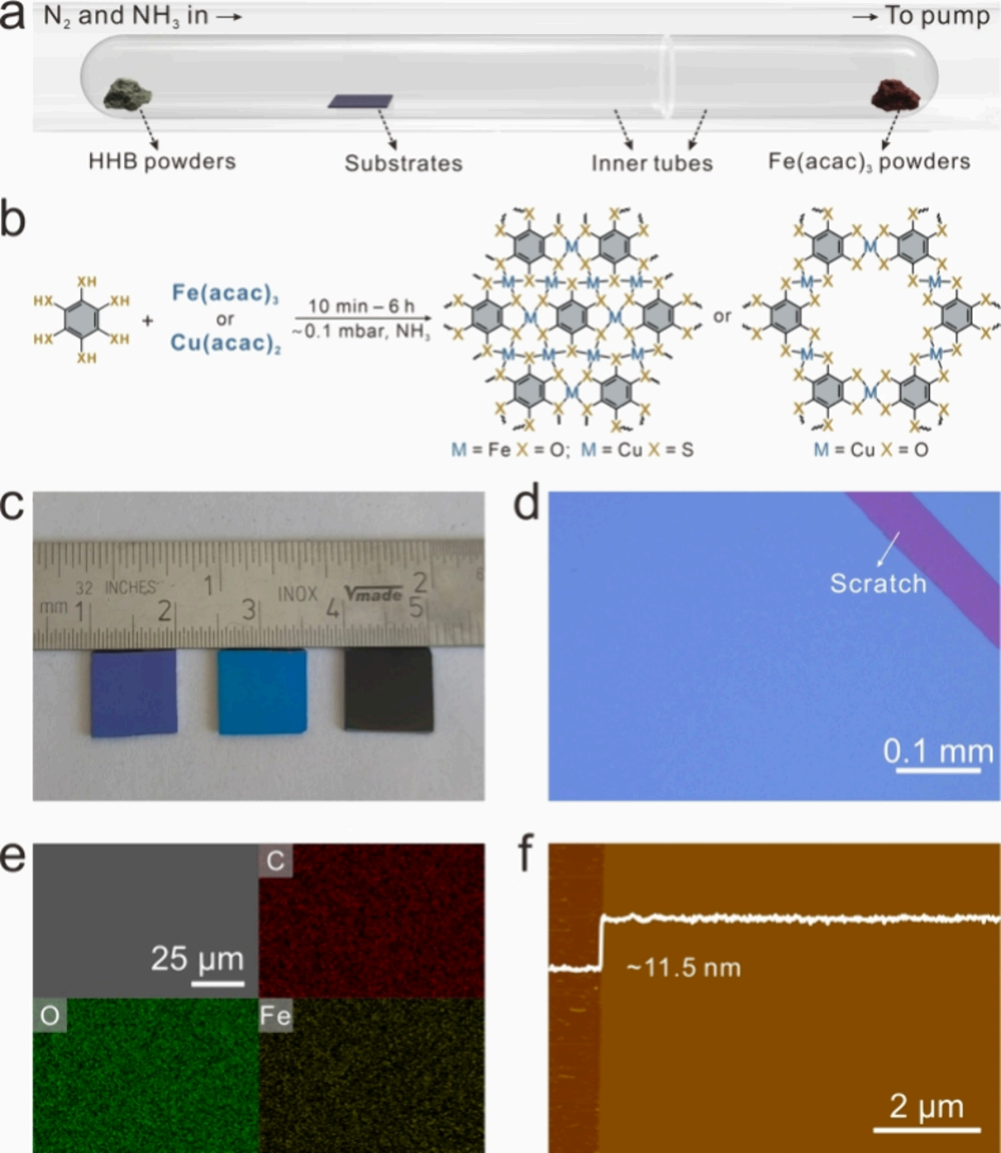
(a) Illustration of the typical CVD setup to perform the NH_3_-assisted growth process. (b) Reaction scheme illustrating the NH_3_-assisted synthesis of 2D c-CPs via coordination between HHB or BHT and Fe­(acac)_3_ or Cu­(acac)_2_. (c) Digital photography image, (d) OM image, (e) SEM image and corresponding EDX elemental mapping, and (f) AFM image of the as-grown **Fe-HHB-w** thin films.

To investigate the crystalline nature of the **Fe-HHB-w** film, scraped fragments were transferred onto copper grids through dispersion and drop-casting and further characterized by transmission electron microscopy (TEM). High-resolution TEM (HR-TEM) images (Figure 3a and Figure S8) reveal a well-preserved periodic structure throughout the entire area (∼10^4^ nm^2^), with no discernible defects observed within the highly ordered single-crystal hexagonal lattice. The corresponding fast Fourier transform (FFT) image exhibited a typical hexagonal diffraction pattern with a (100) plane distance of ∼6.6 Å, which matches well with the 2D Kagome lattice structure of the on-surface Fe-HHB nanodomains.^26^ It is worth noting that the observed hexagonal structure is different from the 3D cubic structure of the in-solution synthesized Fe-THQ powders.^27,28^ We temporarily attribute this discrepancy to the different synthesis environments (vapor phase vs solution phase), which could profoundly affect the topological structures and packing modes. For comparison, the crystallinity of the **Fe-HHB-o** samples synthesized in the absence of NH_3_ was also examined. The sample exhibits smaller crystal domains (∼10^2^ nm^2^) with significant misorientation and large amorphous regions, indicating notably degraded film crystallinity (Figure 3b). As a result of the nearly 100-fold reduction in domain size, the electrical conductivity (σ) of **Fe-HHB-o** thin films decreases by approximately 3 orders of magnitude (∼2 × 10^–3^ S/cm) relative to **Fe-HHB-w** (∼3 S/cm), underscoring the critical role of crystallinity in determining charge transport properties (Figure S9).

**Figure 3:**
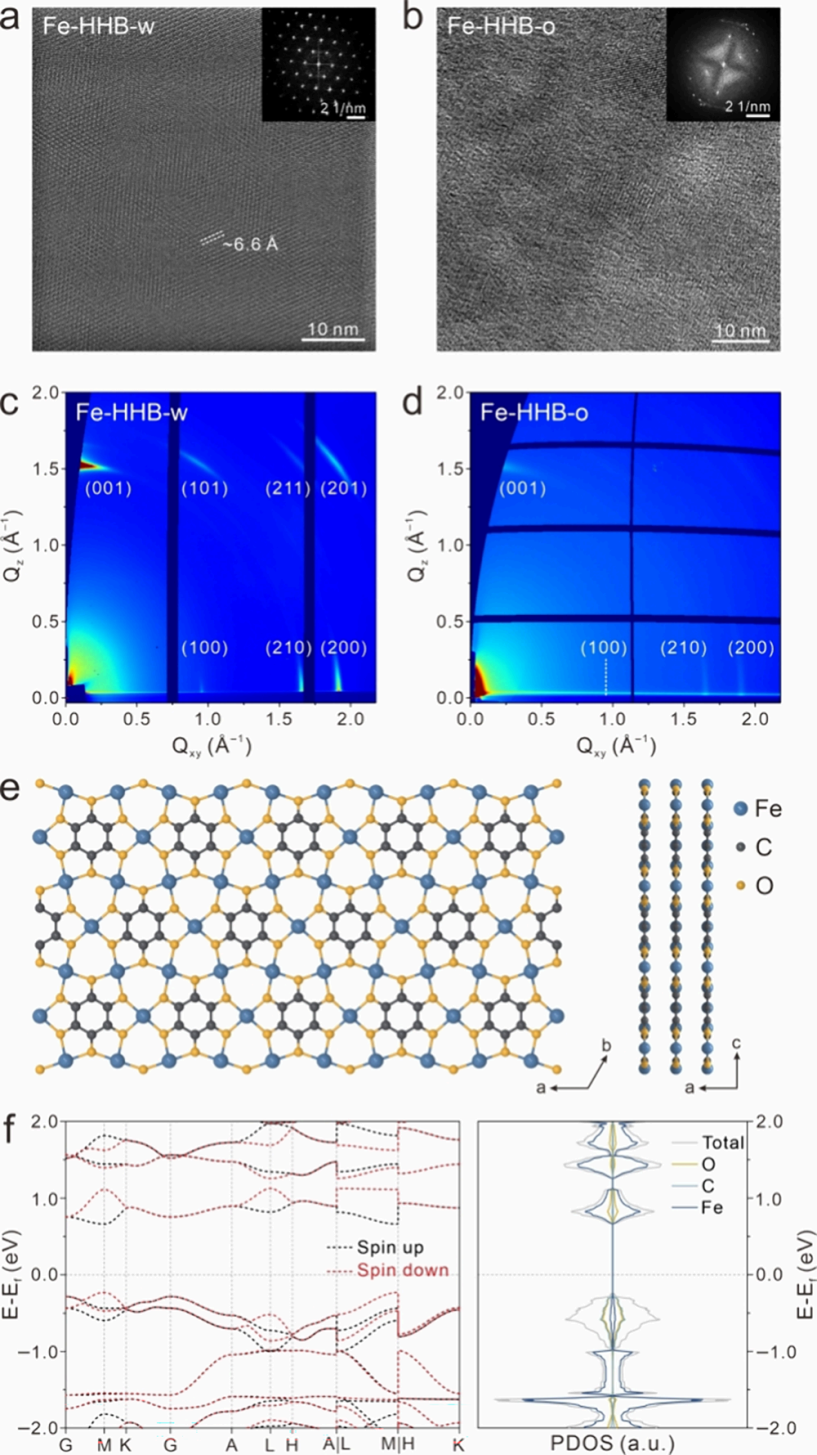
HR-TEM images acquired from (a) **Fe-HHB-w** and (b) **Fe-HHB-o** films. Insets show the corresponding FFT patterns. GIWAXS patterns of (c) **Fe-HHB-w** and (d) **Fe-HHB-o** films, with the corresponding peak assignments, were annotated. (e) AA-stacking model of the hexagonal Fe-HHB with a typical 2D Kagome lattice structure. (f) Calculated band structure of hexagonal Fe-HHB and the corresponding projected density of states (PDOS) for iron (blue), carbon (green), and oxygen (yellow). The Fermi energy was set to 0 eV.

Grazing-incidence wide-angle X-ray scattering (GIWAXS) measurements were also performed to characterize the crystallinity and analyze the lattice structure of the CVD thin films on a macroscopic scale. The 2D GIWAXS pattern of **Fe-HHB-w** shows more diffraction peaks with stronger signals compared to that of **Fe-HHB-o** with similar thickness (Figure 3c,d), indicating the formation of a film with inferior quality without the presence of NH_3_. The significantly improved crystallinity enables a more detailed structural analysis based on the abundant crystal lattice information extracted from the GIWAXS pattern. As shown in Figure 3e, a 2D Kagome lattice structure with an AA-stacking model was established and then optimized by density functional theory (DFT) calculations (Figure S10), evidencing a typical hexagonal unit cell with lattice parameters of *a* = *b* = 7.57 Å. The three in-plane peaks observed at *Q*_*xy*_ = 0.96, 1.66, and 1.92 Å^–1^ in Figure 3c can then be assigned to the (100), (210), and (200) planes of the hexagonal Fe-HHB lattice, respectively. The out-of-plane peak located at *Q*_*z*_ = 1.53 Å^–1^ corresponds to the (001) plane along the π–π stacking direction, which evidences a pronounced face-on layer orientation of the **Fe-HHB-w** film. Notably, synthesizing 2D CP and MOF films with face-on orientations is crucial for investigating their in-plane electronic transport properties. Compared to **Fe-HHB-w**, the broadening of the full width at half-maximum (fwhm) of the GIWAXS peaks extracted from **Fe-HHB-o** also suggests a reduced crystal domain size (Figure S11). The face-on oriented nature of the as-grown film enables us to observe the π–π stacking peak in the powder X-ray diffraction (PXRD) spectrum,^29^ which also reveals a broadened (001) peak in **Fe-HHB-o** samples (Figure S12).

The simulated GIWAXS result with a perfect face-on orientation matches the experimental pattern of **Fe-HHB-w** (Figure S13), thus validating the reliability of the proposed Fe-HHB lattice structure. Furthermore, the obtained selected area electron diffraction (SAED) pattern and the FFT pattern (inset of Figure 3a) of **Fe-HHB-w** along the [001] zone axis align well with the simulated electron diffraction pattern (Figure S14). The FFT pattern extracted from the HR-TEM image of the [100] zone axis was also consistent with the simulated pattern (Figure S15), thus further confirming the accuracy of the proposed Fe-HHB structure from the perspective of electron diffraction. To gain deeper insight into the electronic structure of Fe-HHB, spin-polarized DFT calculations based on its AA-stacking pattern were performed. As shown in Figure 3f, Fe-HHB possesses a direct bandgap of 0.89 eV with interlayer antiferromagnetic ordering. In addition, the ultraviolet-visible (UV–vis) spectrum presents an optical bandgap of ∼1.3 eV (Figure S16), which also suggests its semiconducting feature.

The protons of HHB molecules would act as hindrances to obstruct the coordination bonding with Fe atoms owing to the electrostatic repulsion.^24^ The deprotonation effect of NH_3_ molecules effectively activates HHB molecules in the gas-phase reaction environment, thereby reducing the energy barrier for forming Fe–O coordination bonds and allowing for the smooth formation of ordered coordination networks. The X-ray photoelectron spectroscopy (XPS) analysis (Figure S17) of C 1s reveals a marked increase in the proportion of the C=O component in **Fe-HHB-o** compared to **Fe-HHB-w**, which is most likely due to a rise in uncoordinated O atoms during the formation of **Fe-HHB-o**. In addition, to exclude the possibility that the differences between **Fe-HHB-w** and **Fe-HHB-o** were induced by NH_3_ doping, **Fe-HHB-o** films were treated in the optimal NH_3_ atmosphere but showed no signs of increased conductivity (Figure S18).

To investigate the competing effect of NH_3_, Fe-HHB thin films were synthesized under varying partial pressures of NH_3_ gas. The statistical analyses of σ and (001) peak widths exhibit a similar bell-shaped trend as the NH_3_ concentration rises (Figure S19), suggesting that amplifying the concentration beyond the optimal level could instead degrade the sample quality. The coordination between Fe with excess NH_3_ could result in film quality deterioration, where the uncoordinated HHB molecules tend to self-assemble into crystals, as evidenced by the visible PXRD signals attributed to HHB (Figure S20). To further demonstrate the competing coordination effect of NH_3_, the freshly synthesized **Fe-HHB-w** thin film was exposed to a high-concentration NH_3_ atmosphere. The pronounced morphological changes and the presence of the N element on the film prove the reaction between NH_3_ with the Fe-HHB coordination networks (Figure S21). The XPS N 1s spectrum revealed the presence of Fe-NH_3_ coordination complexes and NH_4_^+^ species (Figure S22), thus confirming the role of NH_3_ as an effective competing coordination species to regulate the formation and breakage of the Fe-HHB coordination bonds. Moreover, the roles of NH_3_ in facilitating ligand deprotonation and serving as a competing coordination species are also applicable to the growth of Cu-HHB (Figures S23 and S24) and Cu-BHT (Figures S25 and S26) thin films with improved quality, as proven by the narrowing of PXRD peak width. The electrical conductivities of the Cu-HHB and Cu-BHT thin films were also increased from ∼51 and ∼595 to ∼113 and ∼905 S/cm, respectively, after adopting the NH_3_-assisted strategy, validating the universality of the proposed strategy.

The high compatibility of the proposed CVD strategy with the device integration technologies ensures the easy fabrication of devices based on the as-grown films with large-area uniformity (Figure S27). To investigate the electrical properties of as-grown Fe-HHB films, four-probe devices were fabricated to measure the temperature-dependent σ. The results shown in Figure 4a revealed that the resistivity of both **Fe-HHB-w** and **Fe-HHB-o** negatively correlates with temperature (ranging from 295 to 573 K), consistent with their semiconducting nature. The data for **Fe-HHB-w** fits well with the Arrhenius equation (Figure 4b and Supporting Information), evidencing thermally activated transport behavior. Meanwhile, for **Fe-HHB-o** (Figure 4c), the linear correlation between ln σ and Τ^–1/4^ suggests that charge transport within the film follows the variable range hopping (VRH) model, possibly originating from the presence of extensive amorphous regions.

**Figure 4:**
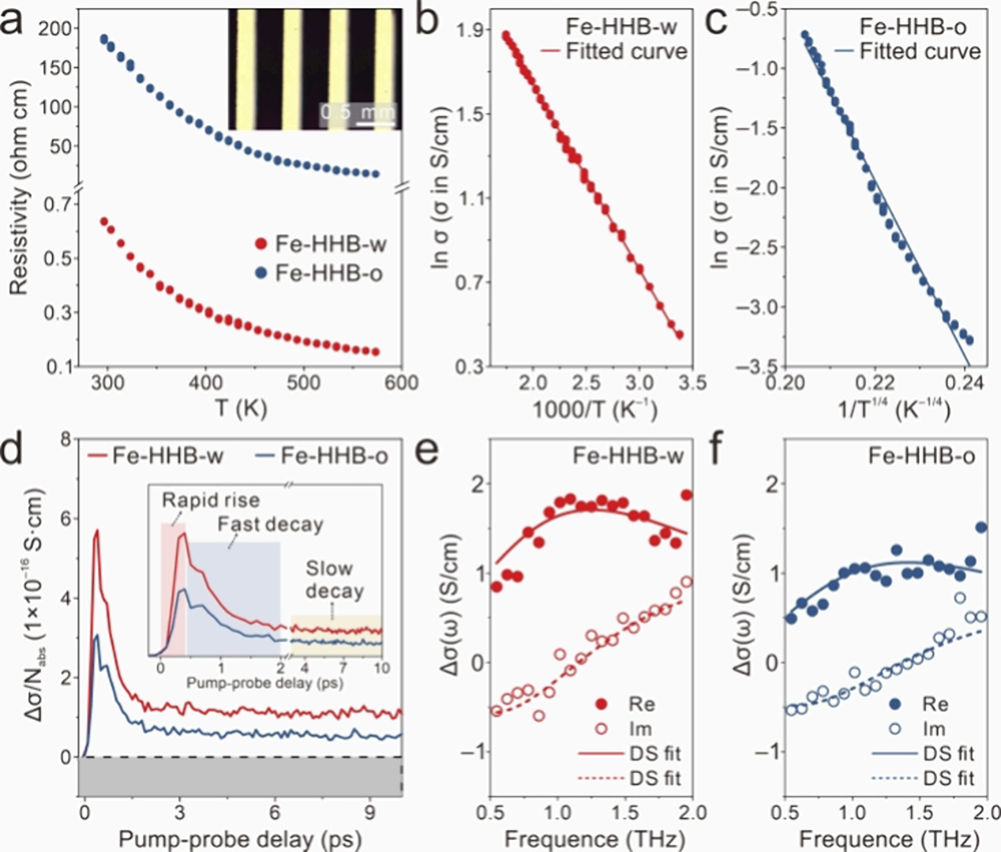
(a) Temperature-dependent electrical resistivity of the **Fe-HHB-w** and **Fe-HHB-o** thin films. The inset shows the OM image of the as-fabricated four-probe device. (b) Plot of ln σ versus Τ^–1^ for **Fe-HHB-w**. (c) Plot of ln σ versus Τ^–1/4^ for **Fe-HHB-o**. (d) THz photoconductivity dynamics of **Fe-HHB-w** and **Fe-HHB-o** thin films normalized by *N*_abs_. The inset shows the optimized plot of the same data, labeling the three distinct components of rapid rise, fast decay, and slow decay. The samples were photoexcited by an 800 nm laser pulse with a pump fluence of ∼1.3 mJ/cm^2^. The frequency-resolved complex photoconductivity of (e) **Fe-HHB-w** and (f) **Fe-HHB-o** was measured at ∼0.5 ps after the maximum photoconductivity. The solid and hollow points are the real and imaginary components, respectively, and the solid and dashed lines represent the fitted DS curves.

Optical-pump THz-probe (OPTP) spectroscopy was utilized as an all-optical, noncontact approach to elucidate the microscopic charge transport properties of **Fe-HHB-w** (∼99 nm thick) and **Fe-HHB-o** (∼101 nm thick) thin films (Figure S28).^30^ During the measurements, an ultrashort (∼50 fs) 800 nm laser pulse optically injects charge carriers into the material by activating interband optical transitions. Subsequently, a time-delayed single-cycle THz pulse with a duration of ∼1 ps interacts with the photogenerated charge carriers and interrogates their photoconductivity in a time- or frequency-resolved manner. As shown in Figure 4d, the photoconductivity (Δσ) of **Fe-HHB-w** and **Fe-HHB-o** was recorded as a function of pump–probe delay. Both samples exhibit a rapid rise in Δσ, reaching their maximum values on the subpicosecond time scale due to the quasi-instantaneous injection of mobile carriers. The photoconductivity then decays biexponentially, with a fast decay component vanishing within ∼2 ps, followed by a slow decay component that persists without significant intensity attenuation for ∼10 ps. The two decay components can be tentatively assigned to charge trapping and electron–hole recombination.^31^ Compared with **Fe-HHB-o**, **Fe-HHB-w** exhibits higher Δσ normalized by absorbed photon density (*N*_abs_). Considering that Δσ*/N*_abs_ = *e*φμ (*e* is the elementary charge and μ is the charge mobility), it can be inferred that **Fe-HHB-w** has a higher μ than **Fe-HHB-o**, assuming that they have similar φ.^32,33^

To gain more quantitative insights, the frequency-resolved complex photoconductivity of both samples was measured. Their spectral dispersions (Figure 4e,f) display a suppressed positive real part and a negative imaginary part at low frequencies, hallmarks of spatially confined charge transport as described by the Drude–Smith (DS) model (see the Supporting Information for a detailed discussion).^34^ In the DS model, a backscattering parameter, *c* quantifies the degree of spatial confinement, with values ranging from −1 (preferential backscattering) to 0 (completely random momentum scattering). The best fits to the data yield scattering times (τ) of 116 ± 7 and 109 ± 8 fs and *c* values of −0.86 and −0.94 for **Fe-HHB-w** and **Fe-HHB-o**, respectively. The nonzero *c* values are consistent with the polycrystalline nature of the samples, where grain boundaries can contribute to backscattering that impedes long-range charge transport. Their charge mobilities in the dc limit (μ_dc_) were estimated to be 31 ± 2 and 12 ± 1 cm^2^/(V·s), following μ_dc_ = *e*τ/m*­(1 + *c*), where *e* is the elementary charge and m* (m* = 0.95 *m*_0_) is the effective mass. The higher μ_dc_ of **Fe-HHB-w** can be attributed to the combined effect of a slightly increased τ and a reduction in backscattering events, likely due to suppressed charge scattering at grain boundaries. This aligns with the improved crystallinity observed in **Fe-HHB-w**, emphasizing that NH_3_ plays a nontrivial role in improving the crystallinity and charge transport properties. The remarkable enhancement of electrical conductivities in **Cu-HHB-w** and **Cu-BHT-w** thin films (referring to the Cu-HHB and Cu-BHT samples grown by the NH_3_-assisted strategy, respectively) is fundamentally attributed to the improved crystallinity, which generally promotes efficient charge transport.

Nanoindentation tests were then performed to evaluate the mechanical properties of Fe-HHB. As shown in the typical load–displacement curves (Figure 5a), the indentation depth of **Fe-HHB-w** was consistently lower than that of **Fe-HHB-o** under the same applied load, indicating a greater resistance to deformation. This is a plausible consequence of improved crystallinity, as the crystalline regions offer greater structural integrity and resistance to dislocation movement. Ensured by the high dissociation energy of Fe–O bonds, an elastic modulus of ∼43.8 GPa (Figure 5b) and an average hardness of ∼2.0 GPa (Figure 5c) were extracted for **Fe-HHB-w** thin films, significantly surpassing those of **Fe-HHB-o** (average elastic modulus of ∼25.7 GPa and hardness of ∼0.9 GPa), **Cu-HHB-w**, **Cu-BHT-w**, and the reported traditional MOFs (Figure 5d and Figure S29).^35^ The superior mechanical performance makes **Fe-HHB-w** thin films suitable for electronic applications requiring wear-resistant active materials such as high-durability wearable devices.

**Figure 5:**
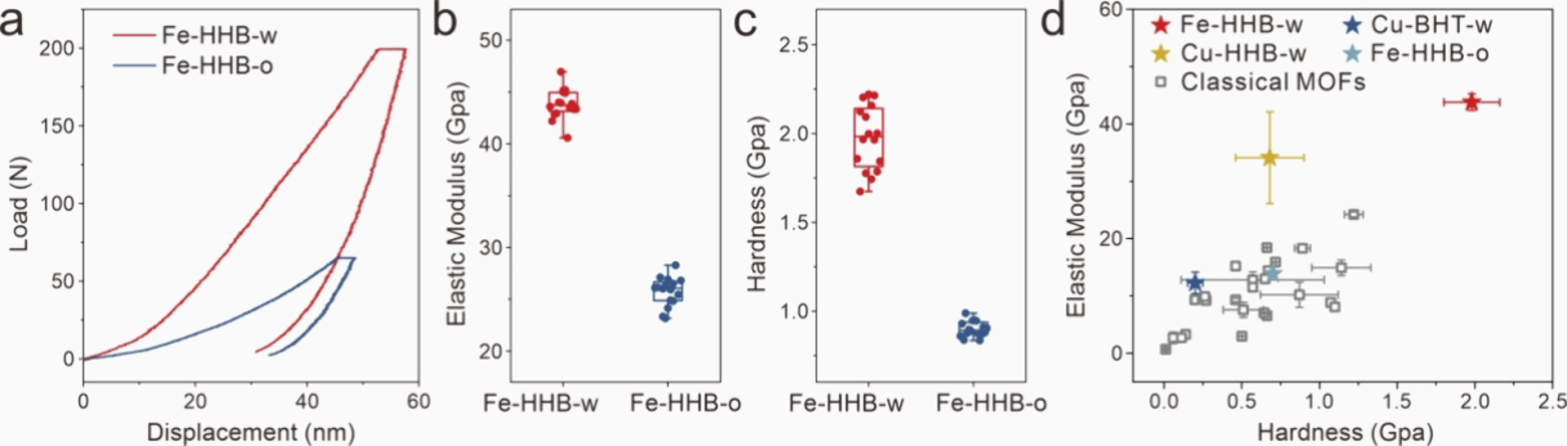
(a) Load–displacement curves of the **Fe-HHB-w** and **Fe-HHB-o** thin films. Statistical analysis of the (b) elastic modulus and (c) hardness of the two samples. (d) Property map of 2D c-CPs and MOFs showing the elastic modulus versus hardness. The CVD 2D c-CP thin films are labeled by stars with varied colors. The classical MOFs are labeled by gray rectangles. See Figure S29 for details.

## Conclusions

In conclusion, we have demonstrated that leveraging the deprotonation effect and coordination competing effect of NH_3_ can significantly enhance the crystallinity of as-grown 2D c-CP samples, enabling the formation of Fe-HHB, Cu-HHB, and Cu-BHT thin films with noticeably elevated electrical and mechanical properties. In addition to universally producing high-quality thin films with large-area uniformity, our method also shows high compatibility with typical device integration technologies, laying a foundation for exploring the fascinating properties of 2D c-CPs and advancing their practical applications. We believe that our proposed synthetic strategy affords feasible access to operational 2D conductive CP and MOF thin films and provides the opportunity to develop CP/MOF electronics.

## Supplementary Material



## Notes

The Supporting Information is available free of charge on the ACS Publications Web site.
General information on methods and characterizations, additional discussion of Arrhenius fitting and OPTP measurements, and supplemental figures (PDF)
