# Supplementary material for: Ammonia-Assisted Chemical Vapor Deposition Growth of Two-Dimensional Conjugated Coordination Polymer Thin Films
Source: J Am Chem Soc. 2025 May 15;147(21):18190–6. doi: 10.1021/jacs.5c04515 (PMC12123600; doi:10.1021/jacs.5c04515)
Supplement: Supplementary file 1 [file ja5c04515_si_001.pdf]

Supporting Information

Ammonia-assisted chemical vapor deposition growth of two-dimensional conjugated coordination polymer thin films

Jinxin Liu[1,‡], Shuai Fu[2,3,‡], Yubin Fu[1,2,‡], Yunxu Chen[1], Kian Tadayon[4], Mike Hambsch[5], Darius Pohl[6], Ye Yang[2], Alina Müller[2], Fengxiang Zhao[7,8], Stefan C. B. Mannsfeld[5], Lei Gao[3], Mischa Bonn[3], Xinliang Feng[1,2,], Renhao Dong[7,8,*]*

[1]*Max Planck Institute for Microstructure Physics, 06120 Halle (Saale), Germany.*
[2]*Center for Advancing Electronics Dresden (CFAED) & Faculty of Chemistry and Food Chemistry, TUD Dresden University of Technology, 01062 Dresden, Germany.*
[3]*Max Planck Institute for Polymer Research, Mainz 55128, Germany.*
[4]*Fraunhofer Institute for Ceramic Technologies and Systems (IKTS), 01109 Dresden, Germany.*
[5]*Center for Advancing Electronics Dresden (CFAED) and Faculty of Electrical and Computer Engineering, TUD Dresden University of Technology, 01062 Dresden, Germany.*
[6]*Dresden Center for Nanoanalysis (DCN), Center for Advancing Electronics Dresden (CFAED), TUD Dresden University of Technology, 01069 Dresden, Germany.*
[7]*Department of Chemistry, The University of Hong Kong, Hong Kong 999077, China.*
[8]*Materials Innovation Institute for Life Sciences and Energy (MILES), HKU-SIRI, Shenzhen 518048, China.*
‡These authors contributed equally to this work.
*To whom correspondence should be addressed: E-mail: xinliang.feng@tu-dresden.de, rhdong@hku.hk

Methods and Characterizations

Treatments of substrates. Before growth, the SiO_2/Si substrates (300-nm SiO_2, MicroChemicals GmbH) and quartz substrates (MicroChemicals GmbH) were cleaned by alternately sonicating in acetone, ethanol and deionized (DI) water for 20 min, and finally dried in a stream of N_2 gas.

Preparation of the container filled with $NH_3 \cdot H_2O$ solution. $NH_3 \cdot H_2O$ solutions with concentrations ranging from 1.4 mmol/L to 14 mol/L (diluted from 25% Ammonia solution, VWR International GmbH) were filled into suitable containers (such as glass vials with silicone septum in the cap or plastic centrifuge tubes). The small opening is created by inserting a syringe needle (0.8 mm in diameter, B. Brown Melsungen AG) into the sealed container.

CVD growth of 2D c-CPs thin films. The CVD growth of 2D c-CP thin films is carried out in a quartz tube furnace (TG3, Carbolite Gero GmbH & Co. KG) and based on a face-to-face inner tube system,[1] as schemed in Figure S1. The growth process of Fe-HHB is: (1) 2 mg HHB powders (HHB = hexahydroxybenzene, TCI Deutschland GmbH) were put into a longer test tube (16 mm in diameter and 160 mm in length) and 20 mg $Fe(acac)_3$ powders ($Fe(acac)_3$ = iron(III) acetylacetonate, BLD Pharmatech GmbH) were put into a shorter test tube (16 mm in diameter and ~65 mm in length), target substrates were then placed in the longer tube with distance of ~70 mm from HHB powder. (2) placing the two test tubes and the $NH_3 \cdot H_2O$ solution container at suitable positions of the quartz tube furnace; (3) purging by 200 sccm N_2 and then pumping the system into low-pressure (~0.5 mbar), NH_3 vapor was then introduced into the system from the $NH_3 \cdot H_2O$ solution container; (3) elevating the temperature of the two heating zones to 200 °C (for HHB) and 105 °C (for $Fe(acac)_3$) in 5 min, respectively; (4) dwelling in 10 min to 6 hours for achieving films with diverse thicknesses; (5) cooling down to room temperature before stopping the pump. For the synthesis of Cu-HHB, $Cu(acac)_2$ powders ($Cu(acac)_2$ = copper(II) acetylacetonate, Sigma-Aldrich Chemie GmbH) were employed as the reactants and heated at 120 °C during the growth process. The growth of Cu-BHT follows the synthesis process described in our previous work.[2]

Characterizations. The AFM topographic images were recorded in the NX10 system (Park Systems) via non-contact mode at room temperature. Raman spectra and mappings were performed in a confocal Raman microscope with an excitation laser wavelength of 532 nm (Alpha300R, WITec) at room temperature. OM images were acquired from an optical microscope (Microscope Axioscope 5, ZEISS) and the Raman microscope. The four-probe electrical conductivity was measured by a SIGNATONE Pro4 system. For the temperature-dependent electrical conductivity measurements, the temperature was controlled by a hot plate (Hei-Tec, Heidolph Instruments GmbH & CO. KG), and the samples were measured under a nitrogen atmosphere to prevent oxidation. To ensure accurate test results, the sample was tested six times at each temperature. The SEM images and corresponding EDX results were recorded by a Zeiss Gemini 500 SEM system. The TEM images were recorded using a JEOL JEM F200 operated at 200 kV acceleration voltage and equipped with a GATAN OneView CMOS camera for fast imaging. Illumination conditions have been set such that, the material do not alter during imaging due to radiolysis. Here, the TEM samples were physically scraped from the Fe-HHB films grown on the substrates and transferred onto a copper grid. Diffraction pattern simulations have been carried out using Tempas 3.0.39 from Total Resolution LLC. A sample thickness of 20 nm, an acceleration voltage of 200 kV and a small convergence of 0.2 mrad was assumed for the calculation of the integrated diffraction pattern. The samples were transferred to an ultrahigh vacuum chamber (ESCALAB 250Xi by Thermo Scientific, base pressure: 2×10^{-10} mbar) for UPS and XPS measurements.

GIWAXS measurements. The GIWAXS measurements of Fe-HHB-w were performed at Beamline ID10 at ESRF, Grenoble, France. The beam energy was 22 keV and the size of the beam was 35 µm (vertical) × 10 µm (horizontal). A Dectris Pilatus 300k area detector was positioned 418.8 mm behind the sample. The sample-detector distance and the beam center on the detector were verified by measuring lanthanum hexaboride and silver behenate as references. The incidence angle of the beam was 0.09° and the sample was exposed for 10 s to the beam. The GIWAXS measurements of Fe-HHB-o were performed at beamline SIRIUS at SOLEIL, St. Aubin, France. The energy of the beam was 10 keV and the beam had dimensions of 70 µm vertically and 500 µm horizontally. A Dectris Pilatus 1M area detector was placed 304.5 mm behind the sample stage and the distance as well as the beam center on the detector were verified using silver behenate. The incidence angle of the beam was 0.12° and six images with 30 s exposure each were recorded.

All the images were analyzed using WxDiff.[3]

Optical pump-THz probe (OPTP) spectroscopy. OPTP spectroscopy was employed to measure the time-resolved and frequency-resolved photoconductivity of Fe-HHB films supported on fused silica substrates. Ultrashort laser pulses with a duration of ~ 50 fs, a central wavelength of 800 nm, and a repetition rate of 1 kHz were generated by a commercial regeneratively amplified and mode-locked titanium:sapphire femtosecond laser system. The generated ultrashort laser pulses were split into different branches for photoexcitation, THz generation, and THz sampling, respectively. Using a pair of (110)-oriented zinc telluride (ZnTe) crystals, THz radiation in the range of ~0.5-2.0 THz was produced and detected via optical rectification and electro-optical sampling methods, respectively. The measurements were performed at room temperature under a dry N_2-purged environment to eliminate THz absorption by water vapor in the air. We obtained the THz photoconductivity ($\Delta\sigma$) of the samples by applying the thin-film approximation to the measured relative attenuation of the THz electric field ($-\Delta E/E$).[4] The obtained photoconductivity is equal to the product of absorbed photon density (N_{abs}), photon-to-free carrier conversion ratio (φ), elementary charge (e), and charge mobility (μ), following $\Delta\sigma = N_{abs} \cdot \varphi \cdot e \cdot \mu$. Given that Fe-HHB-w and Fe-HHB-o have the same chemical nature, it is reasonable to assume that their φ are the same. Therefore, $\Delta\sigma$ divided by N_{abs} ($\Delta\sigma/N_{abs}$) provides a direct comparison of μ. The frequency-resolved complex photoconductivity (σ_{DS}) was described by the Drude-Smith (DS) model, which provides a phenomenological description of spatially confined free carrier transport by considering the backscattering effect:

$$\sigma_{DS} = \frac{\varepsilon_0 \omega_p^2 \tau}{1 - i\omega\tau}\left(1 + \frac{c}{1 - i\omega\tau}\right)$$

where ε_0 is the vacuum permittivity, ω_p is the plasma frequency, τ is the momentum scattering time, and c is the backscattering parameter that assesses the degree of spatial confinement, with values ranging from –1 to 0. For c = –1, the charge carriers are subject to complete backscattering; For c = 0, the model reverts to the classical Drude model describing free carrier transport with completely random momentum scattering.

Fitting of temperature-dependent electrical conductivity data. The temperature-dependent electrical conductivity (σ) data of Fe-HHB thin films synthesized with the presence of NH_3 (named as Fe-HHB-w) was fitted based on the Arrhenius equation:

$$\sigma = \sigma_o \times e^{\frac{-E_a}{k_B T}}$$

where σ_o is a pre-exponential factor, E_a is the activation energy, k_B is the Boltzmann's constant, and T is absolute temperature. A linear correlation can be thereby derived as:

$$ln\sigma = -\frac{E_a}{k_B T} + ln\sigma_o$$

An E_a of ~ 0.08 eV was obtained from analyzing the fitted curve.

Nanoindentation measurements. Quasistatic nanoindentation experiments were conducted on two thin film samples using a Hysitron/Bruker TI950 TriboIndenter to determine hardness and reduced elastic modulus. A Berkovich diamond indenter tip was employed for these measurements. Load-controlled feedback was chosen, with a load function consisting of loading, holding, and unloading phases, each set to 5 seconds. Peak loads were 200 µN for Fe-HHB-w and 65 µN for Fe-HHB-o, resulting in penetration depths of approximately 60 nm and 45 nm, respectively. These depths were kept below 10% of the coating thickness (600 nm) to minimize the influence of the substrate on the measured mechanical properties of the coatings. The load–displacement data were analyzed using the Oliver and Pharr method[5] to derive the reduced elastic modulus and hardness values. To ensure statistical robustness, a grid of 16 indents was systematically performed across each sample.

Theoretical Calculation. Density functional theory (DFT) calculations were carried out using the Vienna ab-initio Simulation Package (VASP)[6,7] version 6.3.1. The electronic wave-functions were expanded in a plane-wave basis set with a kinetic energy cutoff of 520 eV. The geometry optimization convergence was set to forces acting on the ions were smaller than 0.02 $eV \cdot A^{-1}$. Electron-ion interactions were described using the projector augmented wave (PAW) method.[8,9] Generalized gradient approximation (GGA)[10] of the exchange-correlation energy in the form of Perdew-Burke-Ernzerhof (PBE) was applied.[11] We used DFT+U approach to describe the localized *d*-orbitals of Fe ion. The effective Coulomb (U) and exchange (J) terms were set to 4 and 1 eV, respectively,[12] such approach was already successfully applied for similar systems.[13]

Monkhorst-Pack Gamma-centered grid[14] with 6×6×8 dimension was used for K-point sampling of the Brillouin zone for the bulk structure during the geometry optimization. In the computational protocol for the DOS calculation, the K-point grid dimension was changed to 12×12×16. VASPKIT[15] code for pre- and post-processing of the VASP calculated data was used. All the models were subject of full geometry optimization (ionic positions). The effective masses for the electrons and holes were calculated by manually in the case of nearly degenerate electronic states.

Table R1. Table of the dissociation energies of bonds formed by different metals with O and S. Data reproduced from Luo *et al.*[16]

Bonds	Dissociation energies (kJ/mol)
**Fe−O**	**~402-428**
**Fe−S**	~323-329
**Cu−O**	~259-287
**Cu−S**	~274
**Ni−O**	~251-381
**Ni−S**	~344-356
**Co−O**	~241-397
**Co−S**	~331
**Pd−O**	~238-381
**Pd −S**	~385
**Pt−O**	~391
**Pt−S**	~55
**Ag−O**	~221-223
**Ag−S**	~216
**Zn−O**	~159-280
**Zn−S**	~224

We highlight to develop an ammonia-assisted (NH_3-assisted) chemical vapor deposition (CVD) growth strategy using the Fe-HHB (HHB = hexahydroxybenzene) with FeO_4 linkages as an ideal prototype material. Strong metal-ligand coordination bonds such as FeO_4 usually contributes to the irreversible formation of coordination networks, resulting in poorly-crystalized two-dimensional (2D) conductive metal–organic frameworks (MOFs) and conjugated coordination polymers (c-CPs).[17] In our work, we raised this NH_3-assisted synthesis strategy to improve the crystallinity. The presentence of NH_3 was able to facilitate the deprotonation of HHB ligands and meanwhile compete with metal-ligand interactions, which could promote the reversible formation and breakage of the coordination bonds, thereby leading to 2D c-MOF or c-CP thin films with improved crystallinity. With this in mind, the inherently high dissociation energy of the Fe−O bond (Table R1) renders Fe-HHB the most suitable 2D c-CP sample to maximize the beneficial effects of NH_3. That is also the reason why we chose Fe-HHB as the prototype material.

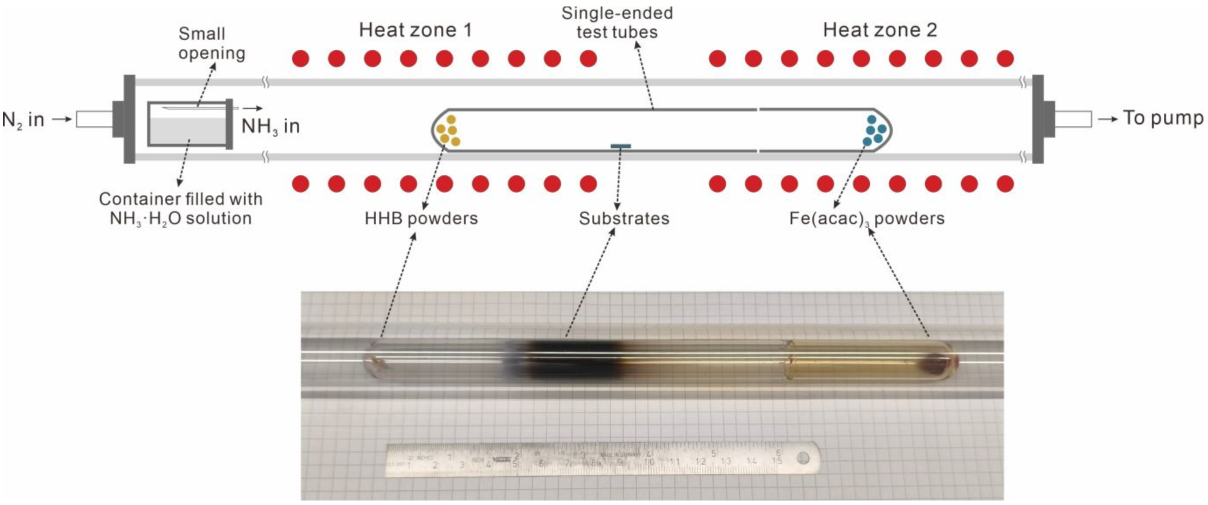

Figure S1. The typical setup for performing the chemical vapor deposition (CVD) growth processes of Fe-HHB with the assistance of NH_3 (named as Fe-HHB-w). The lower panel shows the digital photograph of the tube system after the growth process.

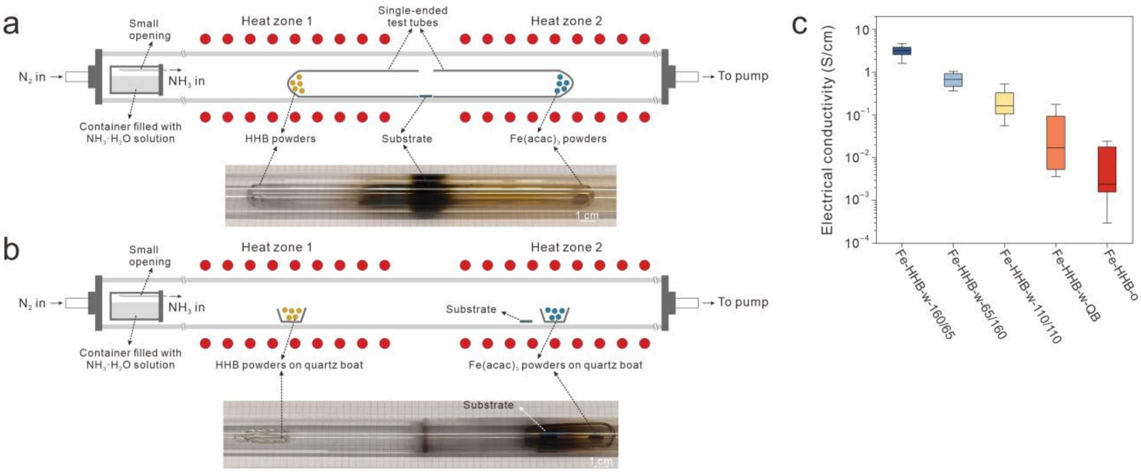

Figure S2. (a) The typical setup for performing the NH_3-assisted CVD growth process based on two single-opening test tubes of equal length. The lower panel shows the digital photograph of the tube system after the growth process. (b) The typical setup for performing the growth based on quartz boats. The lower panel shows the corresponding digital photograph after growth. (c) The electrical conductivities of Fe-HHB-w thin films synthesized with different CVD setups and Fe-HHB-o thin film.

The face-to-face inner tube system could prevent the direct ejection of reactants to the outlet under gas flow.[1] We have thereby adopted a similar configuration and optimized the experimental setup to better align with our equipment and targeted product. Here, we employed a configuration involving two single-opening test tubes placed in direct face-to-face contact, minimizing the gap between them to effectively prevent the loss of vaporized reactants under gas flow conditions. This design aims to enhance the concentration of activated reactants, thereby improving the quality of the synthesized product.[18] Given that the high ligand-to-metal stoichiometric ratio can ensure sufficient chemical potential to overcome the steric hindrance of complete coordination within nanoconfined spaces formed by the intergrowth of crystallines into a continuous thin film, thereby enabling the growth of defect-free CP films.[19] Thus, we utilized a longer single-opening test tube (~160 mm in length) to contain the HHB powders, aiming to retain a higher concentration of HHB ligand (via reducing the loss of vaporized molecules) during the growth. A shorter test tube (~65 mm in length) was then employed to place the $Fe(acac)_3$ powders to fit the length of the heating zones. To further demonstrate the advantages of our CVD setup in synthesizing high-quality Fe-HHB-w thin films, a series of comparative experiments based on different experimental configurations were conducted. First, we employed a reversed configuration, where a longer single-opening test tube (~160 mm in length) was used to contain $Fe(acac)_3$ powders and a shorter one (~65 mm in length) to place HHB powders. As shown in Figure S2c, the resulting Fe-HHB sample after the NH_3-assisted growth process (named as Fe-HHB-w-65/160) exhibited lower electrical conductivity (~ 0.7 S/cm) compared with the Fe-HHB-w thin films grown under the standard configuration (~ 3 S/cm, named as Fe-HHB-w-160/65 here to better differentiate it from other Fe-HHB-w samples), indicating its inferior film quality. We attribute this result to the fact that the vaporized HHB molecules exhibit a higher propensity to escape through the opening of the test tube due to the shorter diffusion distance. Second, we utilized two single-opening test tubes of equal length (~110 mm in length) with HHB and $Fe(acac)_3$ powders placed separately and the substrate positioned at the gap between the tube openings, as schemed in Figure S2a. The corresponding digital photography image of the quartz tube captured after the NH_3-assisted reaction confirmed that the film was formed at the substrate location. However, the resulting thin film (named as Fe-HHB-w-110/110) also exhibited lower electrical conductivity (~ 0.24 S/cm) than the Fe-HHB-w-160/65, further

suggesting suboptimal film quality. We hypothesize that the gap between the openings of the two test tubes serves as a preferential pathway for the escape of reactant molecules. Finally, we have conducted the growth of Fe-HHB without using the face-to-face inner tube system, whereas conventional quartz boats were employed to place the reactant powders, as schemed in Figure S2b. The corresponding digital photography image taken after the NH_3-assisted reaction revealed that the Fe-HHB formation occurred ~20 mm upstream from the $Fe(acac)_3$ powders. The resulting thin film (named as Fe-HHB-w-QB) exhibited the lowest electrical conductivity (~ 0.05 S/cm) among these samples, confirming that the film quality obtained under this configuration was inferior to that achieved using the face-to-face inner tube system.

Moreover, we want to emphasize that all NH_3-assisted Fe-HHB-w samples synthesized under various experimental configurations exhibited significantly higher electrical conductivity compared to the Fe-HHB-o (Fe-HHB-o refers to the sample synthesized in the absence of NH_3) samples (~ 0.002 S/cm). This observation robustly validates the high reliability of our proposed NH_3-assisted growth strategy.

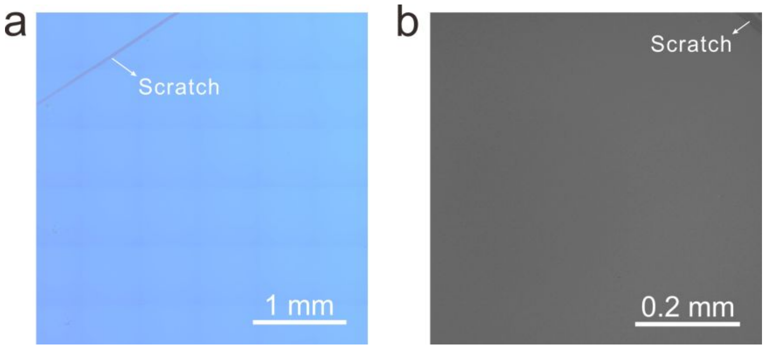

Figure S3. (a) The stitched optical microscope (OM) image and (b) low-magnification scanning electron microscope (SEM) image of as-grown Fe-HHB-w thin films on SiO_2/Si substrates.

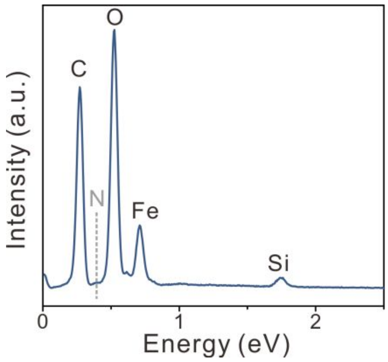

Figure S4. The SEM-based energy-dispersive X-ray spectroscopy (EDX) spectrum of Fe-HHB-w thin films on SiO_2/Si substrates.

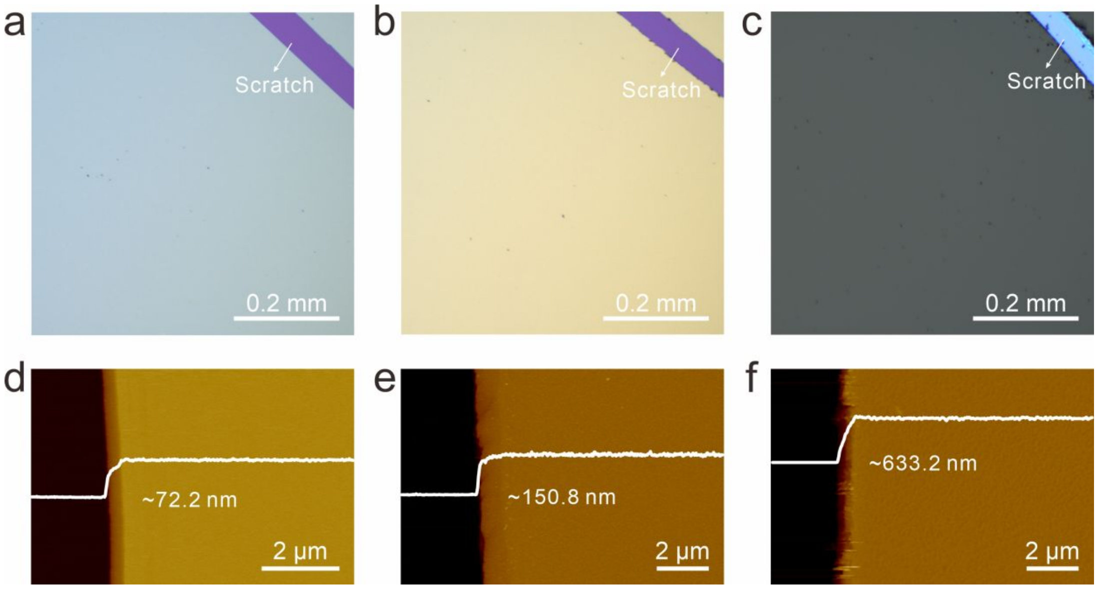

Figure S5. The low-resolution OM image of Fe-HHB-w thin films grown for (a) 0.5 h, (b) 1 h, and (c) 6 h. The corresponding atomic force microscopy (AFM) images of the thin films with thicknesses of (d) ~72.2 nm, (e) ~150.8 nm, and (f) ~633.2 nm.

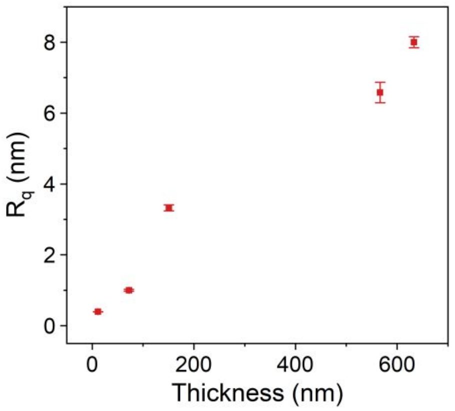

Figure S6. The R_q values of CVD Fe-HHB thin films with different thicknesses.

To quantify the flatness of the CVD Fe-HHB thin films with different growth durations, we have examined the surface roughness (R_q) of these samples through AFM. As presented in Figure S6, the R_q values show a significant increase with increasing the film thickness, where the result aligns with the reported trend of 2D c-CP thin films.[20] In addition, the CVD-grown films exhibit relatively smooth surfaces (R_q value of ~ 8 nm for a 633-nm thick film), with the measured values comparable to those of reported CVD films (R_q value of ~10.7 nm for an 80-nm thick film).[1]

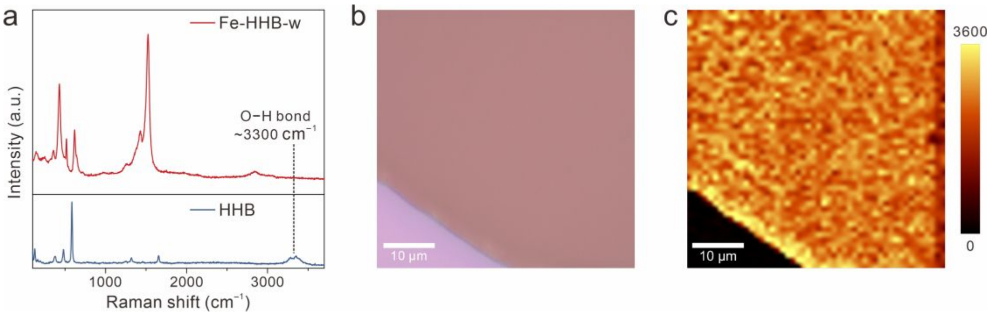

Figure S7. (a) Typical Raman spectra of Fe-HHB-w thin film and HHB powder. The absence of the Raman peak located at ~3300 cm^{-1} (assigned to the O−H bond) confirms the formation of coordination bond between Fe and HHB.[21] (b) OM image of the Fe-HHB-w thin film and (c) the corresponding Raman peak intensity mapping (peak position of 1540 cm^{-1}) with the strength scale on the right. The homogeneous color distribution of the mapping verifies the structural uniformity of the film.

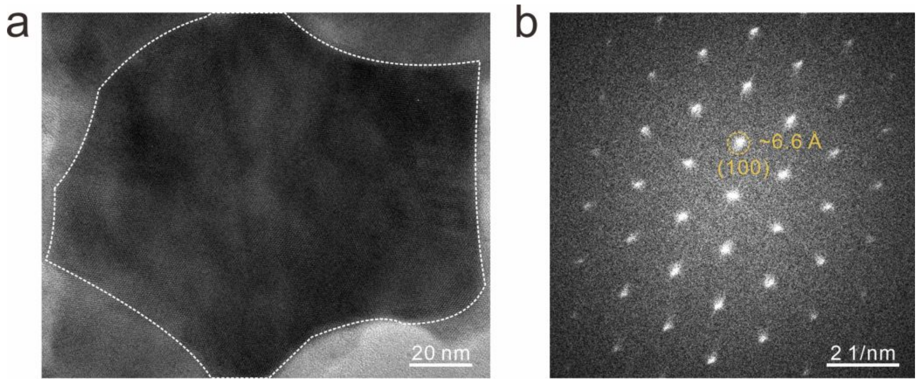

Figure S8. (a) Typical high-resolution transmission electron microscopy (HR-TEM) image of Fe-HHB-w. The single-crystal region (marked by white dash line) is measured as ~11750 nm^2. (b) The fast Fourier transform (FFT) image extracted from (a). Only one set of FFT spots can be observed, confirming the single-crystalline nature of this region. A (100) plane distance of ∼6.6 Å is also indicated.

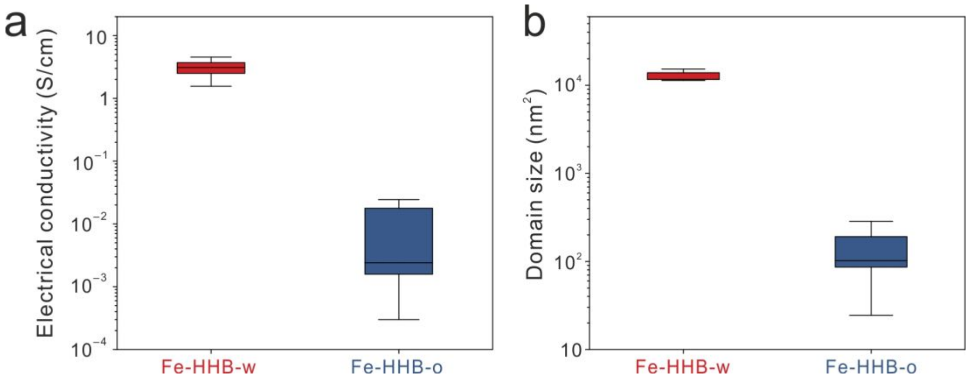

Figure S9. Statistical analysis of the (a) electrical conductivity and (b) domain size of Fe-HHB-w and Fe-HHB-o (Fe-HHB-o refers to the Fe-HHB thin films synthesized without the assistance of NH_3).

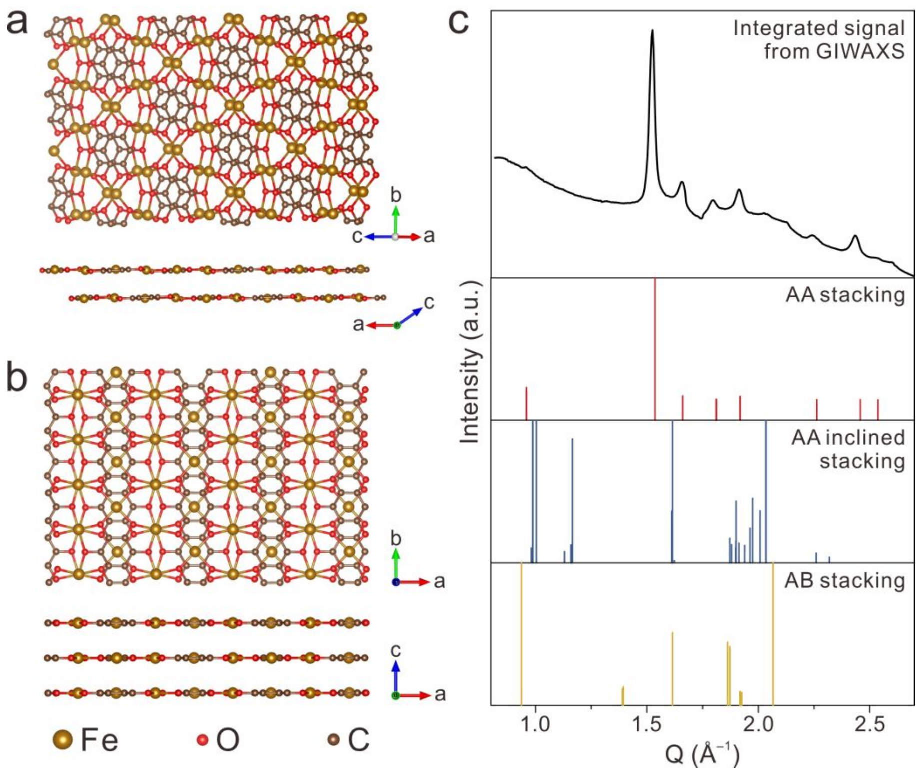

Figure S10. DFT-relaxed Fe-HHB structures with a typical (a) AA-inclined stacking model and (b) AB-stacking model. (c) Integrated line profile from the grazing-incidence wide-angle X-ray scattering (GIWAXS) pattern of Fe-HHB-w thin films and the simulated XRD peak positions (with the unit of $Å^{-1}$) of hexagonal Fe-HHB with different stacking models. The software VESTA is used to draw structures and simulate peak positions.[22]

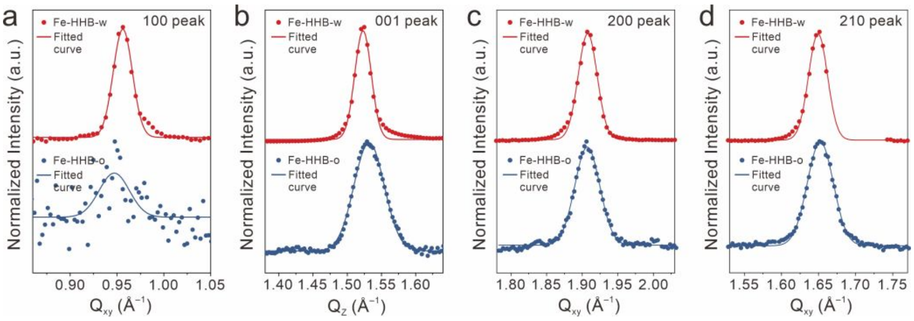

Figure S11. (a) Horizontal linecuts (for the (100) peak) extracted from the GIWAXS patterns of Fe-HHB-w and Fe-HHB-o samples. The full width at half maximum (FWHM) values derived from the fitted curves are 0.020 $Å^{-1}$, assigned to Fe-HHB-w, and 0.033 $Å^{-1}$, assigned to Fe-HHB-o, respectively. (b) The vertical linecuts (for the (001) peak) of the two samples. The resulting FWHM values are 0.024 $Å^{-1}$, assigned to Fe-HHB-w, and 0.044 $Å^{-1}$, assigned to Fe-HHB-o, respectively. The linecuts for the (c) (200) and (d) (210) peaks of the two samples are also analyzed. The resulting FWHM values are 0.026 $Å^{-1}$ and 0.025 $Å^{-1}$, assigned to (200) and (210) peaks of Fe-HHB-w, 0.035 $Å^{-1}$ and 0.036 $Å^{-1}$, assigned to (200) and (210) peaks of Fe-HHB-o, respectively. All these curves were fitted by the Gaussian function.

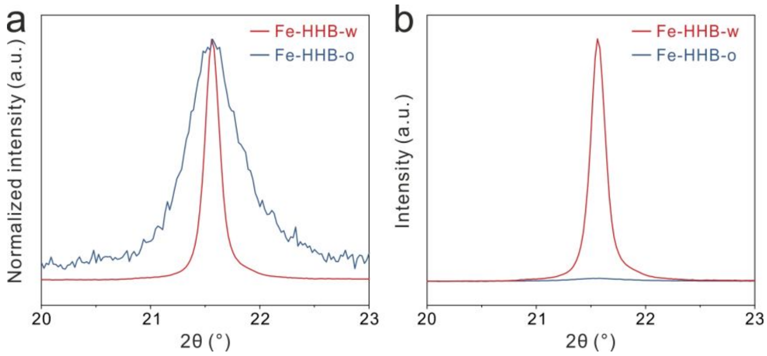

Figure S12. The powder X-ray diffraction (PXRD) spectra to observe the (001) peak of Fe-HHB-w and Fe-HHB-o samples. (a) The spectra plotted with normalized intensity clearly indicate a larger peak width of Fe-HHB-o compared to Fe-HHB-w. (b) The spectra plotted with original intensity clearly indicate a smaller peak height of Fe-HHB-o compared to Fe-HHB-w.

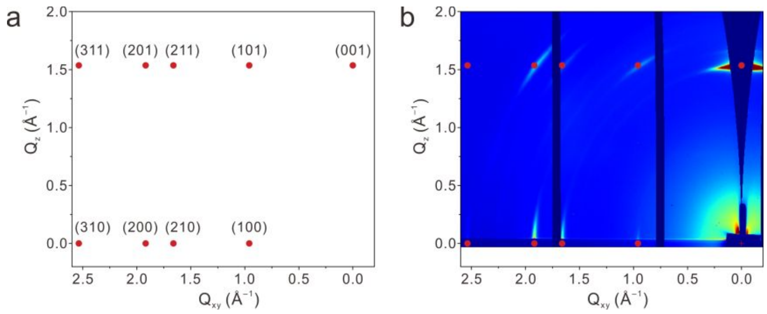

Figure S13. (a) The simulated GIWAXS peak positions of the proposed hexagonal Fe-HHB lattice (γ = 60°) with completely face-on orientation. The software X-Ray Scattering Tools was used for simulating GIWAXS peak positions.[23] (b) Overlaying the simulated peak positions (red dots) on the GIWAXS pattern of Fe-HHB-w, shows almost perfect agreement with experimental signals.

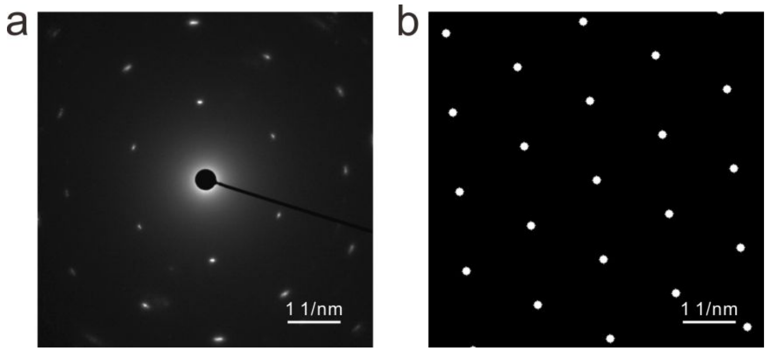

Figure S14. (a) The selected area electron diffraction (SAED) pattern of Fe-HHB-w acquired along the [001] zone axis. (b) The simulated electron diffraction pattern of Fe-HHB along the [001] zone axis. The two patterns show very good consistency.

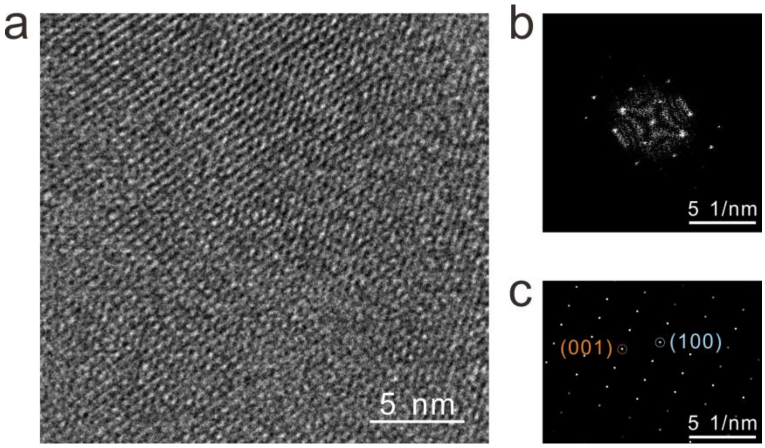

Figure S15. (a) Typical HR-TEM image of Fe-HHB-w acquired along the [100] zone axis. (b) Corresponding FFT pattern of (a). (c) The simulated electron diffraction pattern of Fe-HHB along the [100] zone axis. The two patterns show very good consistency.

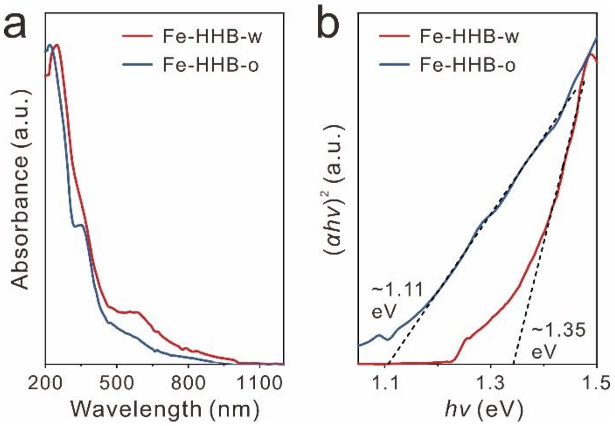

Figure S16. (a) Typical UV–Vis spectroscopy of Fe-HHB-w and Fe-HHB-o thin films directly grown on quartz substrates. (b) The corresponding Tauc plots indicate direct band gap of ~1.35 and 1.11 eV for Fe-HHB-w and Fe-HHB-o, respectively.

The ultraviolet–visible (UV–Vis) absorption spectra of both Fe-HHB-w and Fe-HHB-o exhibit three characteristic absorption bands at approximately 250 nm, 350 nm, and 590 nm. Notably, compared to Fe-HHB-o, the ligand-derived absorption bands at ~250 nm and ~350 nm[24] in Fe-HHB-w display a distinct red shift and spectral broadening, respectively, suggesting enhanced π-d conjugation within the Fe-HHB-w system. Furthermore, the significantly intensified absorption at ~590 nm in Fe-HHB-w, tentatively assigned to ligand-to-metal charge transfer (LMCT) transitions, correlates with its improved crystallinity. This phenomenon can be rationalized by the reduced crystal defects and improved atomic ordering in Fe-HHB-w, which (i) suppress non-radiative energy dissipation pathways during electronic excitations, and (ii) facilitate coherent LMCT processes through enhanced orbital overlap, thereby sharpening the absorption profile and amplifying its intensity. Plotting the absorption data in Tauc coordinates suggests that the optical bandgaps for Fe-HHB-w and Fe-HHB-o are 1.35 and 1.11 eV, respectively. As revealed by the UV–Vis result, the introduction of NH_3 markedly enhances the crystallinity of CVD Fe-HHB thin films. Given that crystal defects can act as charge carrier scattering centers, significantly impeding charge transport and reducing material conductivity, the improvement in crystallinity directly correlates with the observed enhancement in the Fe-HHB's electrical properties. The results also underscored the critical role of the NH_3-assisted growth strategy in optimizing the electrical performance of the 2D c-CP thin films.

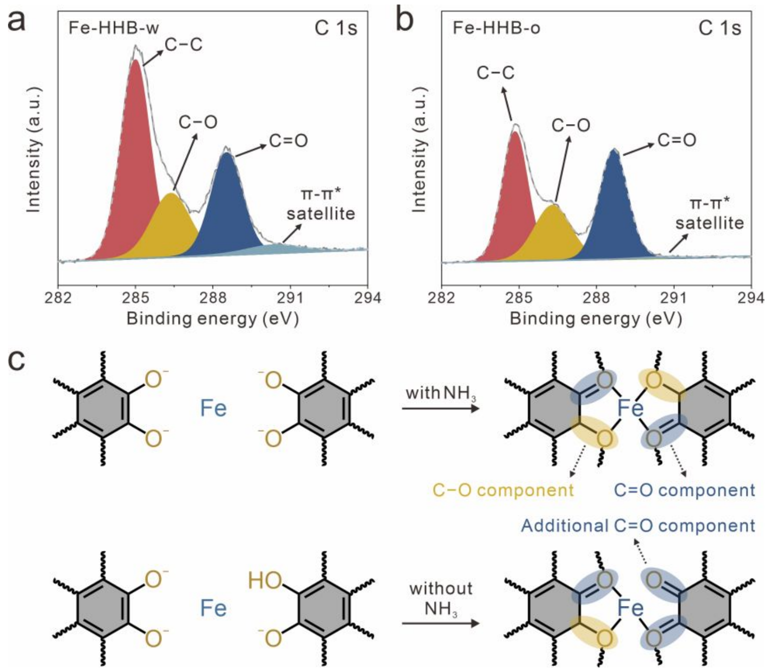

Figure S17. The X-ray photoelectron spectroscopy (XPS) analysis of C 1s component of (a) Fe-HHB-w and (b) Fe-HHB-o. The results reveal a ratio of C=O : C−O of Fe-HHB-w is ~1.28 : 1 and that of Fe-HHB-o is ~1.56 : 1, indicating a marked increase in the proportion of C=O component in Fe-HHB-o compared to Fe-HHB-w. (c) Chemical structures of Fe-HHB coordination networks with and without the presence of NH_3. The overlays highlight the C=O (blue) and C−O (yellow) components. We speculate that the increase of uncoordinated O atoms (with Fe atoms) resulted in the increase of C=O component, which is labeled as additional C=O component in (c).

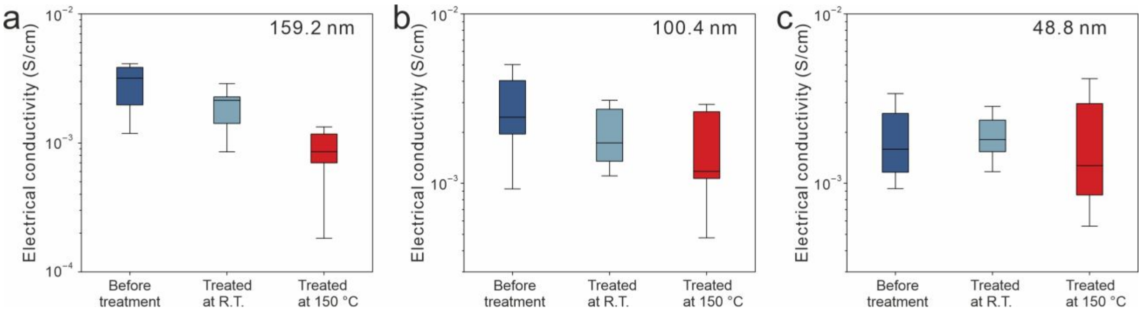

Figure S18. (a) Electrical conductivities of a Fe-HHB-o thin film with a thickness of ~159.2 nm and the same sample after treated in the NH_3 atmosphere (through 0.14 mol/L $NH_3 \cdot H_2O$) at both room temperature (R.T.) and the sample growth temperature (~ 50 °C). Electrical conductivities of the Fe-HHB-o thin films with thickness of (b) ~100.4 nm and (c) ~48.8 nm after the same treatments. No signs of conductivity improvement could be observed after the NH_3 treatments, thus excluding the possibility of NH_3 doping.

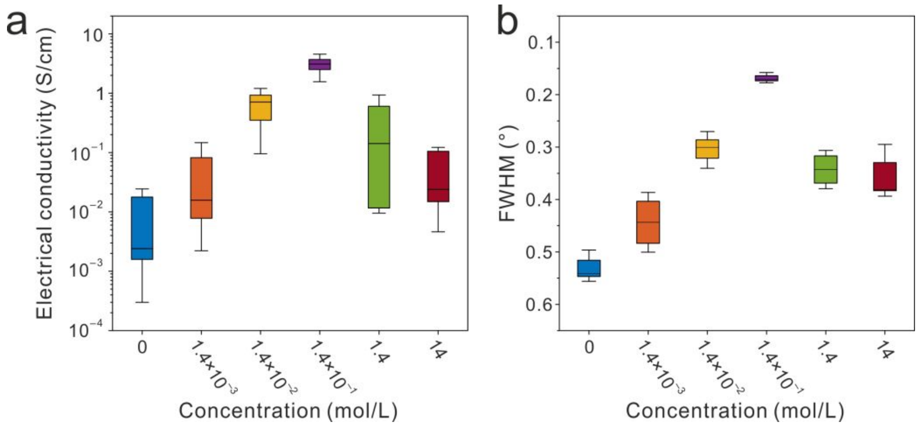

Figure S19. (a) The statistical analysis of the electrical conductivity of the Fe-HHB thin films, which were synthesized under varying $NH_3 \cdot H_2O$ solution concentrations. (b) The statistical analysis of the FWHM values of the (001) peak extracted from the PXRD spectra of these samples. The variation of both electrical conductivity and FWHM exhibit a similarly bell-shaped trend.

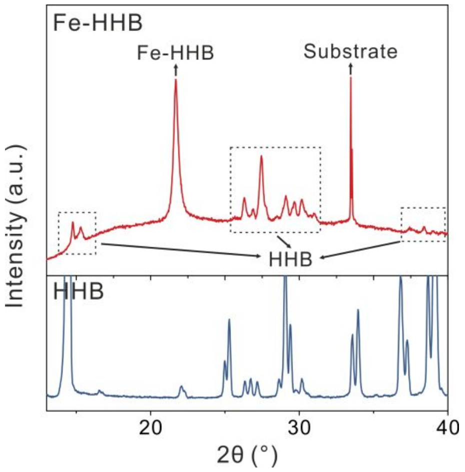

Figure S20. The PXRD spectra of HHB powders (lower panel) and Fe-HHB thin films synthesized with the assistance of excessively supplied NH_3 (upper panel). Visible signals attributed to HHB could be observed in the spectra of this Fe-HHB sample.

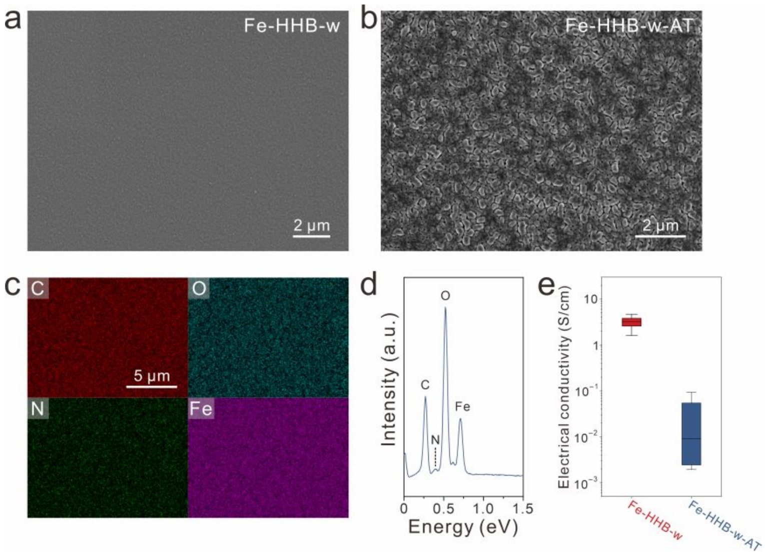

Figure S21. Typical SEM images of (a) freshly-synthesized Fe-HHB-w and (b) Fe-HHB-w after being treated by a high-concentration NH_3 atmosphere (named as Fe-HHB-w-AT), which indicate significant morphological changes after NH_3 treatment. Here, the NH_3 atmosphere was provided by a 14 mol/L $NH_3{\cdot}H_2O$ solution and the Fe-HHB-w sample was maintained at ~50 °C (consistent with the growth temperature of Fe-HHB-w). (c) corresponding EDX elemental mappings and (d) typical EDX spectra acquired from the Fe-HHB-w-AT sample, where the presence of N element was confirmed. (e) Room temperature electrical conductivity of Fe-HHB-w and Fe-HHB-w-AT.

The result suggests that the NH_3 gas induced a pronounced corrosive and decomposing effect on the sample. We attribute this to NH_3 acting as competitive ligands, disrupting the coordination networks of Fe-HHB and breaking them into smaller fragments or molecules, which were subsequently carried away by the gas flow, leaving behind the observed pinholes.

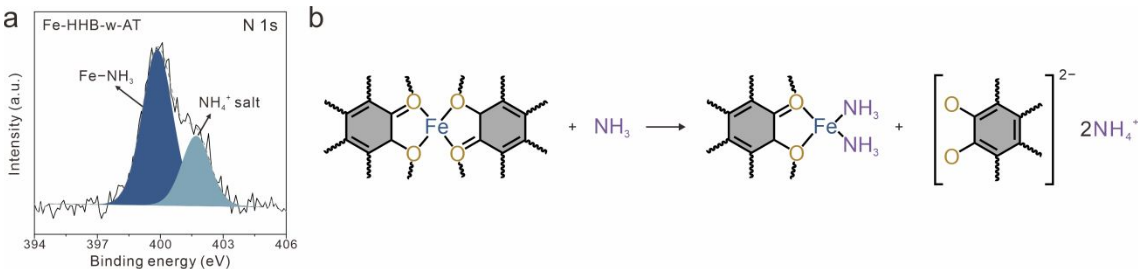

Figure S22. (a) The XPS N 1s spectrum of Fe-HHB-w-AT, which indicates the presence of two different N components within the sample: Fe−NH_3 coordination complexes and NH_4^+ species. (b) A scheme illustrates the possible reaction pathway between FeO_4 linkages of Fe-HHB with NH_3.

The deconvoluted N 1s spectrum of Fe-HHB-w-AT exhibits two characteristic peaks centered at ~399.7 eV and ~401.8 eV (Figure S22a), which indicates the presence of two different N environments within the sample. The peak located at ~399.7 eV can be attributed to the coordination of NH_3 and Fe atoms,[25,26] while another component at ~401.8 eV is commonly associated with the ammonium (NH_4^+) species.[27,28] As suggested by the XPS results, high-concentration NH_3 can deconstruct the original FeO_4 linkages of Fe-HHB, substituting the HHB molecules as the primary ligand in coordination with Fe atoms, as depicted in the reaction scheme in Figure S22b. Thus, we propose that the introduction of NH_3 not only facilitates ligand deprotonation, increasing the concentration of active HHB molecules, but also forms coordination complexes with Fe center, competing with the ligand-metal interactions to promote the reversible formation and breakage of FeO_4 linkages, thereby improving the crystallinity of the final product. In addition, high ligand-to-metal stoichiometric ratio ensures sufficient chemical potential to overcome the steric hindrance of complete coordination within nanoconfined spaces formed by the intergrowth of crystallines into a continuous thin film, thereby enabling the growth of defect-free CP films.[19] The coordination of NH_3 with Fe atoms lowers the availability of active metal species, thereby increasing the ligand-to-metal ratio and ultimately enhancing the crystallinity of the synthesized Fe-HHB thin films.

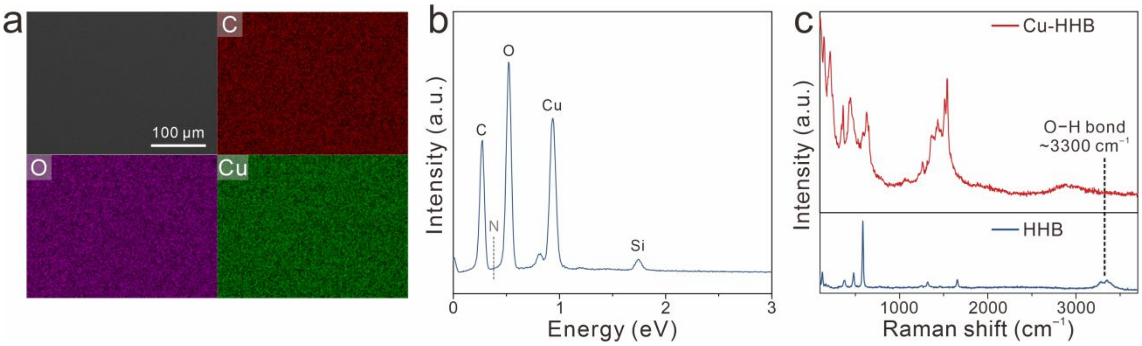

Figure S23. (a) SEM image and the corresponding EDX mapping of Cu-HHB-w thin film. (b) Typical EDX spectra of the sample, no N element is detected. (c) Typical Raman spectra of Cu-HHB-w and HHB powder. The absence of the Raman peak located at ~3300 cm^{-1} (assigned to the O−H bond) confirms the formation of coordination bond between Cu and HHB.

For the Cu-HHB thin films grown with the assistance of NH_3 (named as Cu-HHB-w), SEM image and the corresponding EDX mapping confirmed the uniformity of the synthesized film and its homogeneous elemental distribution (Figure S23a). No obvious signal assigned to the N element was detected in the EDX spectra (Figure S23b), suggesting that no unintended doping occurred from NH_3. The disappearance of the signal assigned to the O−H bond at ~3300 cm^{-1} in the Raman spectra of Cu-HHB-w suggests the efficient coordination between Cu and substitution group (Figure S23c), confirming the formation of coordination networks.

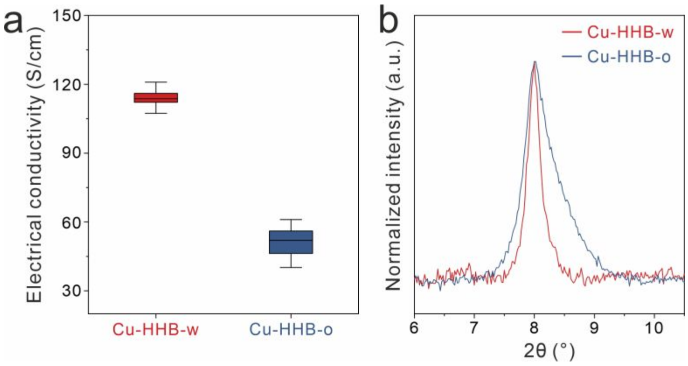

Figure S24. (a) Statistical analysis of the electrical conductivity of Cu-HHB-w and Cu-HHB-o. (b) The PXRD spectra to observe the (100) peak of Cu-HHB-w and Cu-HHB-o samples in normalized intensity, which indicate a larger peak width of Cu-HHB-o compared to Cu-HHB-w.

We have then analyzed the electrical conductivity and PXRD spectra of Cu-HHB-w and the as-grown sample without the assistance of NH_3 (named as Cu-HHB-o) for further comparison. As exhibited in Figure S24a, the electrical conductivity of Cu-HHB-w (~113 S/cm) thin films is more than 2 times higher than that of Cu-HHB-o (~51 S/cm), suggesting an improvement in crystallinity of the CVD Cu-HHB thin films with the NH_3-assisted strategy. It is noteworthy that the electrical conductivity of Cu-HHB-w thin film surpasses previously reported values of Cu-THQ (~92.95 S/cm, THQ = tetrahydroxy-1,4-benzoquinone),[1] which exhibits an almost identical structure with Cu-HHB.[29] A decrease in the FWHM of the Cu-HHB (100) peak in the PXRD patterns provides additional evidence that Cu-HHB-w exhibits higher crystallinity than Cu-HHB-o (Figure S24b), thus demonstrating the effectiveness of the strategy in the growth of high-quality Cu-HHB.

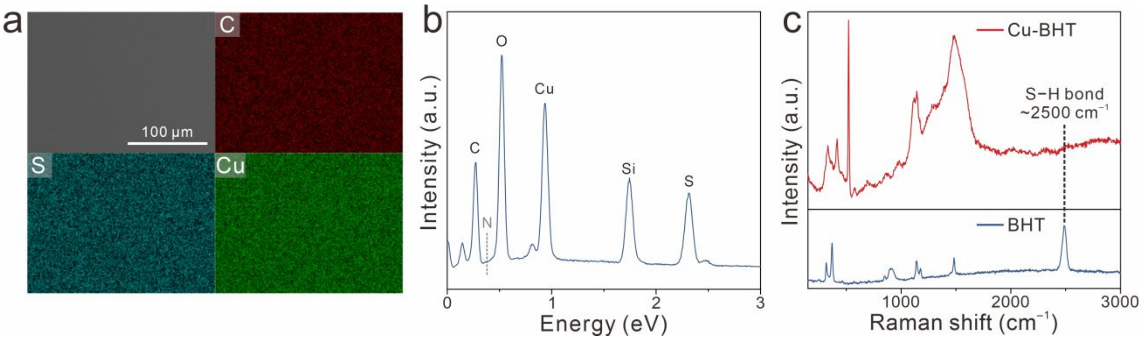

Figure S25. (a) SEM image and the corresponding EDX mapping of Cu-BHT-w thin film. (b) Typical EDX spectra of the sample, no N element is detected. (c) Typical Raman spectra of Cu-BHT-w and BHT powder. The absence of the Raman peak located at ~2500 cm^{-1} (assigned to the S−H bond) confirms the formation of coordination bond between Cu and BHT.

SEM and EDX analyses revealed that the as-grown Cu-BHT (named as Cu-BHT-w) on SiO_2/Si substrate resulted in a highly uniform film with homogeneous elemental distribution (Figures S25a and b). Raman spectroscopy further confirmed the disappearance of the S−H bond signal (~2500 cm^{-1}), indicating the successful formation of coordination CuS_4 linkages (Figure S25c).

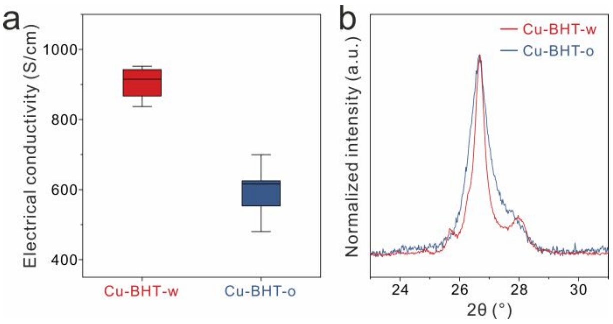

Figure S26. (a) Statistical analysis of the electrical conductivity of Cu-BHT-w and Cu-BHT-o. (b) The PXRD spectra to observe the (001) peak of Cu-BHT-w and Cu-BHT-o samples in normalized intensity, which indicates a larger peak width of Cu-BHT-o compared to Cu-BHT-w.

Comparative studies of electrical conductivities and PXRD spectra (Figures S26a and b) between Cu-BHT-w and sample grown without NH_3 (named as Cu-BHT-o) demonstrated the effectiveness of this strategy in enhancing the crystallinity of Cu-BHT, as evidenced by a 1.5-fold increase in electrical conductivity (from ~595 S/cm to ~905 S/cm) and narrowing of PXRD peaks. The electrical conductivity of Cu-BHT-w thin film directly grown on SiO_2/Si substrate approaches that of Cu-BHT grown on liquid Ga surface (~1007.5 S/cm), which necessitates a transfer procedure.[2] Most importantly, the NH_3-assisted growth strategy enables direct deposition of highly conductive Cu-BHT thin films onto diverse substrates (including SiO_2/Si, quartz, and flexible polymer substrates), eliminating the transfer-related issues such as residual contamination and device fabrication complexities.

Compared to Fe−O bonds, Cu−O and Cu−S bonds generally exhibit lower dissociation energies (Table S1), making the formation of CuO_4 and CuS_4 linkages more reversible. The competitive coordination effect of NH_3 during the growth of Cu-HHB and Cu-BHT is thereby diminished to a certain degree, resulting in less pronounced crystallinity enhancement compared to Fe-HHB. Additionally, the lower activation energy for deprotonation of −SH groups relative to −OH groups[30] further weakens NH_3's role in promoting deprotonation. Consequently, the effectiveness of the strategy in improving crystallinity follows the order: Fe-HHB > Cu-HHB > Cu-BHT. This trend aligns well with our experimental results, further validating the rationale behind the NH_3-assisted growth strategy.

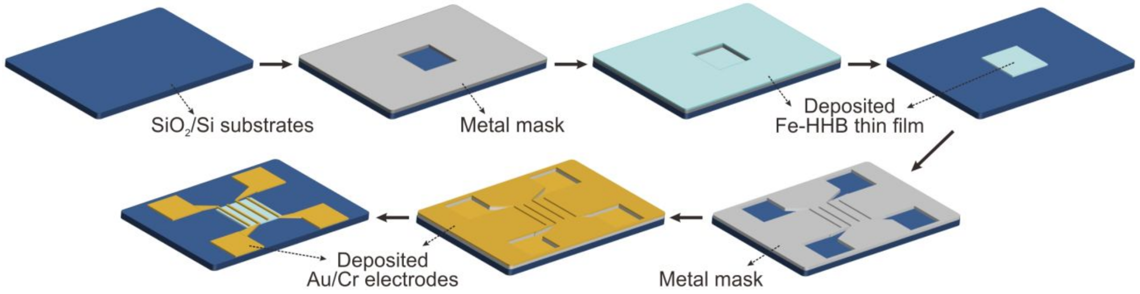

Figure S27. Schematic illustration of the fabrication process of Fe-HHB devices for performing the temperature-dependent electrical conductivity measurements based on the four-probe method.

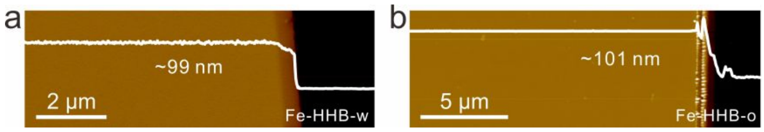

Figure S28. The AFM images of the (a) Fe-HHB-w and (b) Fe-HHB-o thin films for performing the OPTP measurements, which indicate the thickness of the two samples are ~100 nm. The thickness values are significantly smaller than the THz wavelength (1 THz ≈ 300 μm), justifying the use of the thin-film approximation.

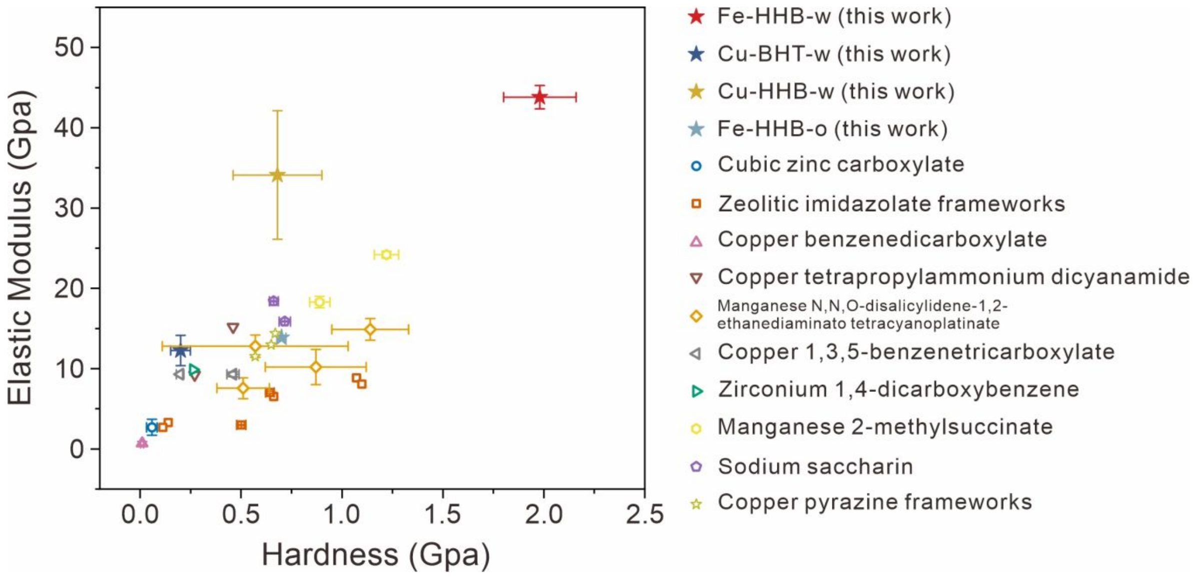

Figure S29. Property map of 2D c-CPs and MOFs showing elastic modulus *versus* hardness. Fe-HHB-w is labeled by a red star. Experimental data of classical MOF materials were collected from published works: cubic zinc carboxylate;[31] zeolitic imidazolate frameworks;[32–35] copper benzenedicarboxylate;[36] copper tetrapropylammonium dicyanamide;[37] manganese N,N,O-disalicylidene-1,2-ethanediaminato tetracyanoplatinate;[38] copper 1,3,5-benzenetricarboxylate;[39,40] zirconium 1,4-dicarboxybenzene;[41,42] manganese 2-methylsuccinate;[43] sodium saccharin;[44] copper pyrazine frameworks.[45]

As exhibited in Figure S29, Fe-HHB-w exhibited the highest hardness (~2.0 GPa) and elastic modulus (~43.8 GPa) among these samples, exceeding those of Cu-HHB-w (~34.1 GPa of elastic modulus and ~0.69 GPa of hardness), Cu-BHT-w (12.2 GPa of elastic modulus and ~0.20 GPa of hardness) and other MOFs. Considering that Fe–O bonds generally have higher bond energy and shorter bond lengths than Cu–O and Cu–S bonds,[16,46] this accounts for the superior mechanical performance of Fe-HHB-w.[47] The high mechanical strength ensures Fe-HHB-w thin films are particularly suitable for the wear-resistant electronics. In addition, the vapor-phase synthesis method at relatively low temperatures (~ 50 °C) is highly compatible with a majority of common flexible substrates, thus making the CVD Fe-HHB-w a promising active material for high-durability wearable devices.

Reference

(1) Choe, M.; Koo, J. Y.; Park, I.; Ohtsu, H.; Shim, J. H.; Choi, H. C.; Park, S. S. Chemical Vapor Deposition of Edge-on Oriented 2D Conductive Metal–Organic Framework Thin Films. *J. Am. Chem. Soc.* **2022**, *144* (37), 16726–16731.

(2) Liu, J.; Chen, Y.; Huang, X.; Ren, Y.; Hambsch, M.; Bodesheim, D.; Pohl, D.; Li, X.; Deconinck, M.; Zhang, B.; Löffler, M.; Liao, Z.; Zhao, F.; Dianat, A.; Cuniberti, G.; Vaynzof, Y.; Gao, J.; Hao, J.; Mannsfeld, S. C. B.; Feng, X.; Dong, R. On-Liquid-Gallium Surface Synthesis of Ultrasmooth Thin Films of Conductive Metal–Organic Frameworks. *Nat. Synth.* **2024**, *3* (6), 715–726.

(3) Mannsfeld, S. C. B.; Tang, M. L.; Bao, Z. Thin Film Structure of Triisopropylsilylethynyl-Functionalized Pentacene and Tetraceno[2,3-b]Thiophene from Grazing Incidence X-Ray Diffraction. *Adv. Mater.* **2011**, *23* (1), 127–131.

(4) Ulbricht, R.; Hendry, E.; Shan, J.; Heinz, T. F.; Bonn, M. Carrier Dynamics in Semiconductors Studied with Time-Resolved Terahertz Spectroscopy. *Rev. Mod. Phys.* **2011**, *83* (2), 543–586.

(5) Oliver, W. C.; Pharr, G. M. Measurement of Hardness and Elastic Modulus by Instrumented Indentation: Advances in Understanding and Refinements to Methodology. *J. Mater. Res.* **2004**, *19* (1), 3–20.

(6) Kresse, G.; Furthmüller, J. Efficient Iterative Schemes for Ab Initio Total-Energy Calculations Using a Plane-Wave Basis Set. *Phys. Rev. B* **1996**, *54* (16), 11169–11186.

(7) Kresse, G.; Furthmüller, J. Efficiency of Ab-Initio Total Energy Calculations for Metals and Semiconductors Using a Plane-Wave Basis Set. *Computational Materials Science* **1996**, *6* (1), 15–50.

(8) Blöchl, P. E. Projector Augmented-Wave Method. *Phys. Rev. B* **1994**, *50* (24), 17953–17979.

(9) Kresse, G.; Joubert, D. From Ultrasoft Pseudopotentials to the Projector Augmented-Wave Method. *Phys. Rev. B* **1999**, *59* (3), 1758–1775.

(10) Perdew, J. P.; Chevary, J. A.; Vosko, S. H.; Jackson, K. A.; Pederson, M. R.; Singh, D. J.; Fiolhais, C. Atoms, Molecules, Solids, and Surfaces: Applications of the Generalized Gradient Approximation for Exchange and Correlation. *Phys. Rev. B* **1992**, *46* (11), 6671–6687.

(11) Perdew, J. P.; Burke, K.; Ernzerhof, M. Generalized Gradient Approximation Made Simple. *Phys. Rev. Lett.* **1996**, *77* (18), 3865–3868.

(12) Anisimov, V. I.; Aryasetiawan, F.; Lichtenstein, A. I. First-Principles Calculations of the Electronic Structure and Spectra of Strongly Correlated Systems: The LDA+ U Method. *J. Phys.: Condens. Matter* **1997**, *9* (4), 767–808.

(13) Yang, C.; Dong, R.; Wang, M.; Petkov, P. St.; Zhang, Z.; Wang, M.; Han, P.; Ballabio, M.; Bräuninger, S. A.; Liao, Z.; Zhang, J.; Schwotzer, F.; Zschech, E.; Klauss, H.-H.; Cánovas, E.; Kaskel, S.; Bonn, M.; Zhou, S.; Heine, T.; Feng, X. A Semiconducting Layered Metal-Organic Framework Magnet. *Nat. Commun.* **2019**, *10* (1), 3260.

(14) Monkhorst, H. J.; Pack, J. D. Special Points for Brillouin-Zone Integrations. *Phys. Rev. B* **1976**, *13* (12), 5188–5192.

(15) Wang, V.; Xu, N.; Liu, J.-C.; Tang, G.; Geng, W.-T. VASPKIT: A User-Friendly Interface Facilitating High-Throughput Computing and Analysis Using VASP Code. *Comput. Phys. Commun.* **2021**, *267*, 108033.

(16) Luo, Y.-R. *Comprehensive Handbook of Chemical Bond Energies*; CRC Press, 2007.

(17) Dou, J. H.; Arguilla, M. Q.; Luo, Y.; Li, J.; Zhang, W.; Sun, L.; Mancuso, J. L.; Yang, L.; Chen, T.; Parent, L. R.; Skorupskii, G.; Libretto, N. J.; Sun, C.; Yang, M. C.; Dip, P. V.; Brignole, E. J.; Miller, J. T.; Kong, J.; Hendon, C. H.; Sun, J.; Dincă, M. Atomically Precise Single-Crystal Structures of Electrically Conducting 2D Metal–Organic Frameworks. *Nat. Mater.* **2021**, *20* (2), 222–228.

(18) Wu, K.-J.; Tse, E. C. M.; Shang, C.; Guo, Z. Nucleation and Growth in Solution Synthesis of Nanostructures – from Fundamentals to Advanced Applications. *Prog. Mater Sci.* **2022**, *123*, 100821.

(19) Liu, G.; Guo, Y.; Chen, C.; Lu, Y.; Chen, G.; Liu, G.; Han, Y.; Jin, W.; Xu, N. Eliminating Lattice Defects in Metal–Organic Framework Molecular-Sieving Membranes. *Nat. Mater.* **2023**, *22* (6), 769–776.

(20) Huang, X.; Sheng, P.; Tu, Z.; Zhang, F.; Wang, J.; Geng, H.; Zou, Y.; Di, C. A.; Yi, Y.; Sun, Y.; Xu, W.; Zhu, D. A Two-Dimensional π-d Conjugated Coordination Polymer with Extremely High Electrical Conductivity and Ambipolar Transport Behaviour. *Nat. Commun.* **2015**, *6* (1), 7408.

(21) Amores, M.; Wada, K.; Sakaushi, K.; Nishihara, H. Reversible Energy Storage in Layered

Copper-Based Coordination Polymers: Unveiling the Influence of the Ligand's Functional Group on Their Electrochemical Properties. *J. Phys. Chem. C* **2020**, *124* (17), 9215–9224.
(22) Momma, K.; Izumi, F. VESTA 3 for Three-Dimensional Visualization of Crystal, Volumetric and Morphology Data. *J. Appl. Crystallogr.* **2011**, *44* (6), 1272–1276.
(23) Venkatesan, N. R.; Kennard, R. M.; DeCrescent, R. A.; Nakayama, H.; Dahlman, C. J.; Perry, E. E.; Schuller, J. A.; Chabinyc, M. L. Phase Intergrowth and Structural Defects in Organic Metal Halide Ruddlesden–Popper Thin Films. *Chem. Mater.* **2018**, *30* (23), 8615–8623.
(24) Liu, Y.; Zhu, X.; Qin, L.; Zhang, T.; Wang, S.; Jin, Y.; Song, L.; Zhang, M.-D. Benzenehexol-Modified $Co_2(OH)_2CO_3$ Nanowire Substrates for Highly Efficient Electrocatalytic Alkaline Hydrogen Evolution. *ACS Appl. Nano Mater.* **2022**, *5* (8), 10596–10602.
(25) Tufts, B. J.; Abrahams, I. L.; Caley, C. E.; Lunt, S. R.; Miskelly, G. M.; Sailor, M. J.; Santangelo, P. G.; Lewis, N. S.; Roe, A. L.; Hodgson, K. O. XPS and EXAFS Studies of the Reactions of Cobalt(III) Ammine Complexes with Gallium Arsenide Surfaces. *J. Am. Chem. Soc.* **1990**, *112* (13), 5123–5136.
(26) Zhou, Y.; Tao, X.; Chen, G.; Lu, R.; Wang, D.; Chen, M.-X.; Jin, E.; Yang, J.; Liang, H.-W.; Zhao, Y.; Feng, X.; Narita, A.; Müllen, K. Multilayer Stabilization for Fabricating High-Loading Single-Atom Catalysts. *Nat. Commun.* **2020**, *11* (1), 5892.
(27) Thompson, Michael.; Nunn, A. D.; Treher, E. N. X-Ray Photoelectron Spectroscopy of Potential Technetium-Based Organ Imaging Agents. *Anal. Chem.* **1986**, *58* (14), 3100–3103.
(28) Nefedov, V. I.; Salyn, Ya. V.; Shtemenko, A. V.; Kotelnikova, A. S. X-Ray Photoelectron Study of *Trans*-Influence of the Re Re Multiple Bond. *Inorg. Chim. Acta* **1980**, *45*, L49–L50.
(29) Park, J.; Hinckley, A. C.; Huang, Z.; Feng, D.; Yakovenko, A. A.; Lee, M.; Chen, S.; Zou, X.; Bao, Z. Synthetic Routes for a 2D Semiconductive Copper Hexahydroxybenzene Metal-Organic Framework. *J. Am. Chem. Soc.* **2018**, *140* (44), 14533–14537.
(30) Kitaguchi, Y.; Habuka, S.; Mitsui, T.; Okuyama, H.; Hatta, S.; Aruga, T. Comparative Study of Phenol and Thiophenol Adsorption on Cu(110). *J. Chem. Phys.* **2013**, *139* (4), 44708.
(31) Bahr, D. F.; Reid, J. A.; Mook, W. M.; Bauer, C. A.; Stumpf, R.; Skulan, A. J.; Moody, N. R.; Simmons, B. A.; Shindel, M. M.; Allendorf, M. D. Mechanical Properties of Cubic Zinc Carboxylate IRMOF-1 Metal-Organic Framework Crystals. *Phys. Rev. B* **2007**, *76* (18), 184106.
(32) Tan, J. C.; Bennett, T. D.; Cheetham, A. K. Chemical Structure, Network Topology, and Porosity Effects on the Mechanical Properties of Zeolitic Imidazolate Frameworks. *Proc. Natl. Acad. Sci.* **2010**, *107* (22), 9938–9943.
(33) Bennett, T. D.; Goodwin, A. L.; Dove, M. T.; Keen, D. A.; Tucker, M. G.; Barney, E. R.; Soper, A. K.; Bithell, E. G.; Tan, J.-C.; Cheetham, A. K. Structure and Properties of an Amorphous Metal-Organic Framework. *Phys. Rev. Lett.* **2010**, *104* (11), 115503.
(34) Bennett, T. D.; Tan, J.-C.; Moggach, S. A.; Galvelis, R.; Mellot-Draznieks, C.; Reisner, B. A.; Thirumurugan, A.; Allan, David. R.; Cheetham, A. K. Mechanical Properties of Dense Zeolitic Imidazolate Frameworks (ZIFs): A High-Pressure X-Ray Diffraction, Nanoindentation and Computational Study of the Zinc Framework $Zn(Im)_2$, and Its Lithium Boron Analogue, $LiB(Im)_4$. *Chem. Eur. J.* **2010**, *16* (35), 10684–10690.
(35) Tian, T.; Velazquez-Garcia, J.; Bennett, T. D.; Fairen-Jimenez, D. Mechanically and Chemically Robust ZIF-8 Monoliths with High Volumetric Adsorption Capacity. *J. Mater. Chem. A* **2015**, *3* (6), 2999–3005.
(36) Huang, C.; Guo, Z.; Zheng, X.; Chen, X.; Xue, Z.; Zhang, S.; Li, X.; Guan, B.; Li, X.; Hu, G.; Wang, T. Deformable Metal–Organic Framework Nanosheets for Heterogeneous Catalytic Reactions. *J. Am. Chem. Soc.* **2020**, *142* (20), 9408–9414.
(37) Ji, L.-J.; Li, Z.-G.; Dong, L.-Y.; Zhang, Y.; Guo, T.-M.; Gao, F.-F.; Feng, G.-Q.; Li, W. Mechanical Properties of a New Hybrid Inorganic–Organic Framework: A Nanoindentation, High-Pressure X-Ray Diffraction, and Computational Study. *Cryst. Growth Des.* **2022**, *22* (12), 6984–6994.
(38) Iwai, Y.; Kusumoto, S.; Suzuki, R.; Tachibana, M.; Komatsu, K.; Kikuchi, T.; Kawaguchi, S. I.; Kadobayashi, H.; Masubuchi, Y.; Yamamoto, Y.; Ozawa, Y.; Abe, M.; Hirai, K.; Le Ouay, B.; Ohba, M.; Ohtani, R. Mechanical Properties of Modulative Undulating Layers in Two-Dimensional Metal–Organic Frameworks. *Chem. Mater.* **2024**, *36* (11), 5446–5455.
(39) Bundschuh, S.; Kraft, O.; Arslan, H. K.; Gliemann, H.; Weidler, P. G.; Wöll, C. Mechanical Properties of Metal-Organic Frameworks: An Indentation Study on Epitaxial Thin Films. *Appl. Phys.*

Lett. **2012**, *101* (10), 101910.
(40) Tian, T.; Zeng, Z.; Vulpe, D.; Casco, M. E.; Divitini, G.; Midgley, P. A.; Silvestre-Albero, J.; Tan, J.-C.; Moghadam, P. Z.; Fairen-Jimenez, D. A Sol–Gel Monolithic Metal–Organic Framework with Enhanced Methane Uptake. *Nat. Mater.* **2018**, *17* (2), 174–179.
(41) Connolly, B. M.; Aragones-Anglada, M.; Gandara-Loe, J.; Danaf, N. A.; Lamb, D. C.; Mehta, J. P.; Vulpe, D.; Wuttke, S.; Silvestre-Albero, J.; Moghadam, P. Z.; Wheatley, A. E. H.; Fairen-Jimenez, D. Tuning Porosity in Macroscopic Monolithic Metal-Organic Frameworks for Exceptional Natural Gas Storage. *Nat. Commun.* **2019**, *10* (1), 2345.
(42) Wu, D.; Huang, X.; Duan, X.; Jiang, Y.; Shi, D.; Chang, L.; Zhang, L.; He, J. Static and Time-Dependent Plasticity of Monolithic Metal–Organic Frameworks. *J. Phys. Chem. C* **2023**, *127* (9), 4749–4758.
(43) Li, W.; Barton, P. T.; Kiran, M. S. R. N.; Burwood, R. P.; Ramamurty, U.; Cheetham, A. K. Magnetic and Mechanical Anisotropy in a Manganese 2-Methylsuccinate Framework Structure. *Chem. Eur. J.* **2011**, *17* (44), 12429–12436.
(44) Kiran, M. S. R. N.; Varughese, S.; Ramamurty, U.; Desiraju, G. R. Effect of Dehydration on the Mechanical Properties of Sodium Saccharin Dihydrate Probed with Nanoindentation. *CrystEngComm* **2012**, *14* (7), 2489–2493.
(45) Li, W.; Kiran, M. S. R. N.; Manson, J. L.; Schlueter, J. A.; Thirumurugan, A.; Ramamurty, U.; Cheetham, A. K. Mechanical Properties of a Metal–Organic Framework Containing Hydrogen-Bonded Bifluoride Linkers. *Chem. Commun.* **2013**, *49* (40), 4471–4473.
(46) Speight, J. *Lange's Handbook of Chemistry, Seventeenth Edition*; McGraw-Hill Education, 2016.
(47) Isotta, E.; Peng, W.; Balodhi, A.; Zevalkink, A. Elastic Moduli: A Tool for Understanding Chemical Bonding and Thermal Transport in Thermoelectric Materials. *Angew. Chem. Int. Ed.* **2023**, *62* (12), e202213649.
